# IFIT1 Differentially Interferes with Translation and Replication of Alphavirus Genomes and Promotes Induction of Type I Interferon

**DOI:** 10.1371/journal.ppat.1004863

**Published:** 2015-04-30

**Authors:** Josephine M. Reynaud, Dal Young Kim, Svetlana Atasheva, Aliaksandra Rasalouskaya, James P. White, Michael S. Diamond, Scott C. Weaver, Elena I. Frolova, Ilya Frolov

**Affiliations:** 1 Department of Microbiology, University of Alabama at Birmingham, Birmingham, Alabama, United States of America; 2 Department of Medicine, Washington University School of Medicine, Saint Louis, Missouri, United States of America; 3 Department of Pathology & Immunology, Washington University School of Medicine, Saint Louis, Missouri, United States of America; 4 Department of Molecular Microbiology, Washington University School of Medicine, St. Louis, Missouri, United States of America; 5 Institute for Human Infections and Immunity, University of Texas Medical Branch, Galveston, Texas, United States of America; 6 Center for Tropical Diseases, University of Texas Medical Branch, Galveston, Texas, United States of America; 7 Department of Pathology, University of Texas Medical Branch, Galveston, Texas, United States of America; The Scripps Research Institute, UNITED STATES

## Abstract

Alphaviruses are a group of widely distributed human and animal pathogens. It is well established that their replication is sensitive to type I IFN treatment, but the mechanism of IFN inhibitory function remains poorly understood. Using a new experimental system, we demonstrate that in the presence of IFN-β, activation of interferon-stimulated genes (ISGs) does not interfere with either attachment of alphavirus virions to the cells, or their entry and nucleocapsid disassembly. However, it strongly affects translation of the virion-delivered virus-specific RNAs. One of the ISG products, IFIT1 protein, plays a major role in this translation block, although an IFIT1-independent mechanism is also involved. The 5’UTRs of the alphavirus genomes were found to differ significantly in their ability to drive translation in the presence of increased concentration of IFIT1. Prior studies have shown that adaptation of naturally circulating alphaviruses to replication in tissue culture results in accumulation of mutations in the 5’UTR, which increase the efficiency of the promoter located in the 5’end of the genome. Here, we show that these mutations also decrease resistance of viral RNA to IFIT1-induced translation inhibition. In the presence of higher levels of IFIT1, alphaviruses with wt 5’UTRs became potent inducers of type I IFN, suggesting a new mechanism of type I IFN induction. We applied this knowledge of IFIT1 interaction with alphaviruses to develop new attenuated variants of Venezuelan equine encephalitis and chikungunya viruses that are more sensitive to the antiviral effects of IFIT1, and thus could serve as novel vaccine candidates.

## Author Summary

Alphaviruses represent a group of highly important human pathogens, which are transmitted by mosquito vectors between vertebrate hosts. Alphavirus replication in vertebrates depends on their ability to interfere with host antiviral responses on both cellular and organismal levels. The identification of cellular factors, which affect virus replication, and characterization of their functions may prove crucial for the design of new effective vaccine candidates. We have demonstrated that the protein product of one of the interferon-stimulated genes, IFIT1, is a potent inhibitor of translation of the incoming alphavirus genomes and ultimately, virus replication. The secondary structure of the 5’untranslated regions (5’UTRs) of alphavirus genomes was shown to play a critical role in alphavirus resistance to this inhibitory effect. Moreover, in IFIT1-expressing cells, wt alphaviruses exhibiting low sensitivity to IFIT1 also were found to induce high levels of type I IFN. Altogether, our data show that alphavirus 5’UTRs were evolutionarily selected to meet the requirements of both functioning as promoters for positive- and negative-strand RNA synthesis and supporting the resistance to inhibitory effects of IFIT1. We further exploited this new knowledge to develop mutated alphaviruses, which displayed higher sensitivity to IFIT1 and more attenuated phenotypes.

## Introduction

The Alphavirus genus in the *Togaviridae* family contains 30 species and has a worldwide distribution [1]. Most alphaviruses are transmitted by mosquito vectors between amplifying vertebrate hosts [2]. In vertebrates, alphaviruses cause acute infections, characterized by high titer viremia that allows infection of mosquitoes during blood feeding. A number of alphaviruses, including Venezuelan (VEEV) and eastern (EEEV) equine encephalitis viruses, and chikungunya virus (CHIKV), are globally important, emerging public health threats. These viruses can cause epidemics of severe meningoencephalitis with frequent lethal outcomes, or polyarthritis with excruciating and chronic joint pain [3]. Over the last ten years, there have been multiple outbreaks of CHIKV infection with millions of people infected [4–7], including an ongoing epidemic in the Americas with more than 40 countries affected and over 1.1 million suspected cases. Epizootic strains of VEEV and EEEV are almost universally lethal for equids [8]. In addition, the latter viruses can be transmitted efficiently by aerosol [9], are highly stable in lyophilized form, and were developed previously as biological warfare agents [9]. In spite of their public health threat, the pathogenesis of alphaviruses on the molecular and cellular levels remains poorly understood, and no approved vaccines or therapies exist for any of them.

Alphavirus infections are sensitive to type I IFN both *in vivo* and *in vitro* [10–12]. Mice defective in IFN signaling succumb to most alphavirus infections within a few days [13]. IFN treatment induces a large set of IFN-stimulated genes (ISGs), whose protein products prevent infection with many pathogens, including alphaviruses [14–17]. Although hundreds of ISGs have been described, only few have had their antiviral functions unambiguously defined, particularly for alphaviruses [18–24]. The involvement of so many cellular genes implies that the antiviral response might be highly redundant against a given pathogen. Such redundancy would reduce the possibility of selection of virus mutants resistant to an ISG product. However, it is also plausible that only a subset of ISGs is essential for protection against a specific pathogen or groups of related pathogens. Thus, identification of pathogen-specific ISGs might lead to development of targeted therapeutics lacking the therapy-limiting side effects of IFNs.

Previous studies have shown that translation of alphavirus-specific, but not cellular RNAs, is inhibited in IFN-β-treated cells [25–28]. However, these studies were based on either using replication-competent viral RNAs or *in vitro*-synthesized RNAs, delivered into the cells by transfection procedures that do not mimic the natural infectious process. To overcome such limitations, we designed a new experimental system, which allows selective investigation of the effect of ISG activation on the very early steps of alphavirus infection, prior to viral RNA replication. To achieve this, we exploited the unique characteristics of the recently discovered Eilat (EILV) alphavirus [29, 30].

Similar to other members of the genus, the EILV genome is an 11.5 kb positive polarity RNA, which is packaged into icosahedral nucleocapsids, surrounded by lipid envelopes with imbedded trimeric glycoprotein spikes [30]. Upon delivery into the cytoplasm, the genomic RNA serves as a template for translation of viral nonstructural proteins, nsP1-4, which mediate replication of the genomic RNA and transcription of the subgenomic (SG) RNA [1]. The latter RNA functions as a template for synthesis of viral structural proteins. EILV retains the critical characteristics of other alphaviruses in terms of its genomic coding strategy, virion structure and nucleotide sequences of the RNA promoters [30]. The amino acid sequences of the replication enzymes and structural proteins also demonstrate high degrees of conservation with other alphaviruses. However, importantly, while the nsP1-4 complexes actively replicate viral RNA in mosquito cells, they are nonfunctional in vertebrate cell lines [29].

We have modified the EILV genome and converted this virus into an efficient system for delivery of nonreplicating RNAs of interest into vertebrate cells via the natural alphavirus-specific entry pathway. Using EIL/VEEV chimeric viruses and other experimental approaches, we studied the components of early, type I IFN-induced cell resistance to alphaviruses and the mechanisms employed by alphaviruses to interfere with the inhibitory effect of type I IFN. Our studies demonstrated the following: (i) type I IFN pretreatment blocks translation of alphavirus RNAs delivered in infectious virions, but does not affect virus attachment, entry or nucleocapsid disassembly; (ii) the IFN-induced inhibition of translation relies on both IFIT1-dependent and-independent mechanisms; (iii) the 5'UTRs derived from genomes of different alphaviruses differ in their ability to drive translation of the downstream genes after type I IFN pretreatment or in the presence of IFIT1; (iv) the efficiency of replication of the corresponding viruses in IFIT1-producing cells correlates directly with translation sensitivity of their genomes to IFIT1; (v) the presence of IFIT1 makes wt, but not tissue culture-adapted, alphaviruses strong inducers of type I IFN; and (vi) by modifying sequences in the 5'UTRs, we have generated alphaviruses with graded levels of sensitivity to IFIT1, an approach that can be applied to vaccine development. Collectively, our results indicate that IFIT1 can act both as an antiviral effector molecule and an inducer of innate immunity against alphaviruses. Our data also provide a mechanistic explanation for alphavirus resistance and sensitivity to IFIT1 and adaptation of natural isolates to cell culture.

## Results

### EIL/VEEV chimeric viruses

One of the distinguishing features of the newly discovered EILV alphavirus is its very efficient replication and plaque formation in *Aedes albopictus* and other mosquito cells yet inability to replicate in cells of vertebrate origin [29]. This cell-restricted phenotype is determined both by structural proteins, which are not able to promote EILV entry into vertebrate cells, and the nonstructural proteins, which appear to lack appropriate interaction with key cellular factors required for RNA replication [29].

**Fig 1:**
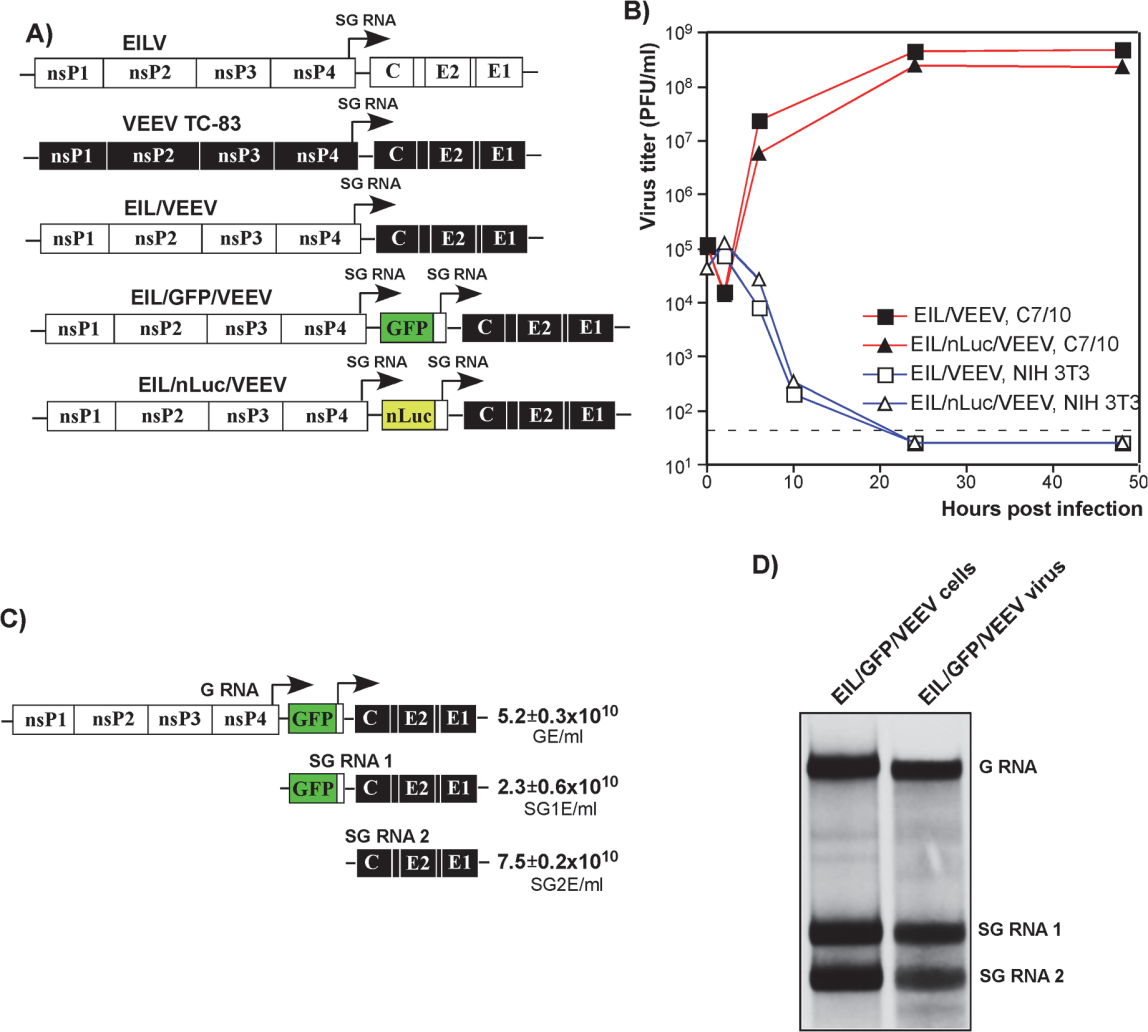
Chimeric EIL/VEEV viruses do not replicate in vertebrate cells, and package subgenomic RNAs into viral particles in mosquito cells. **(A)** The schematic representation of EILV, VEEV TC-83 and recombinant viral genomes. **(B)** Replication of EIL/VEEV and EIL/nLuc/VEEV in C7/10 and NIH 3T3 cells. Cells were infected at an MOI of 20 PFU/cell. Media were harvested at the indicated times post infection, and titers were determined by plaque assay on C7/10 cells. (C) Concentrations of genomic RNA, SG RNA 1 and SG RNA 2 in the sample of EIL/GFP/VEEV harvested at 48 h PI of C7/10 cells. The number of copies per ml of virus were determined by RT-qPCR using primers specific to EILV nsP2, GFP and VEEV E2 genes as described in Materials and Methods. (D) C7/10 cells were infected with EIL/GFP/VEEV at an MOI of 20 PFU/cell, and viral RNA were labeled with [^3^H]uridine between 16 and 24 h post infection. RNAs were isolated from both the cells and the released viral particles and analyzed by agarose gel electrophoresis in denaturing conditions.

We have exploited EILV’s inability to replicate RNA in vertebrate cells to engineer a set of chimeric viruses, in which the EILV structural genes were replaced by those derived from the attenuated VEEV strain TC-83. VEEV TC-83 demonstrates efficient replication in a wide variety of insect and vertebrate cells, and the designed chimeras were expected to infect vertebrate cells but to fail to subsequently replicate their RNA. The EIL/VEEV recombinant virus encoded the EILV replication machinery, and all of the EILV-specific *cis*-acting RNA promoter elements, including the 5' and 3'-UTRs of the viral genome and SG RNA, as well as the 51-nt conserved sequence element (CSE) and SG promoter [1], driving expression of VEEV structural protein genes (**Fig 1A**). Other chimeras, EIL/nLuc/VEEV and EIL/GFP/VEEV, had additional SG promoters to control the expression of nanoLuc (nLuc) and GFP, respectively (**Fig 1A**). In C7/10 mosquito cells, within 24 h post of electroporation with *in vitro*-synthesized RNAs, chimeric viruses were released into the supernatant attaining titers exceeding 5x10^8^ PFU/ml. Almost all of the cells transfected with EIL/GFP/VEEV RNA were GFP-positive within 8 h post electroporation, indicating that no additional adaptive mutations were required for RNA replication in mosquito cells. The generated chimeras were capable of efficient replication in mosquito C7/10 cells (**Fig 1B**), but no virus growth was detected in vertebrate NIH 3T3 (**Fig 1B**), BHK-21, or Vero cells. In contrast to recombinant chimeras, the control VEEV TC-83 productively replicated in both vertebrate and mosquito cell lines (**S1 Fig**).

A lack of infectious virus release does not conclusively rule out the possibility of viral RNA replication. Thus, we next assessed levels of EIL/VEEV- and VEEV TC-83-specific RNAs in the NIH 3T3 cells at different times post infection by RT-qPCR using primers specific to the VEEV E2 structural gene (**S1 Fig**). By 24 h post infection, we detected more than a 10,000-fold increase in the concentration of VEEV TC-83-specific RNAs, compared to input RNA present in the viral particles adsorbed during inoculation. Within the same time frame, EIL/VEEV-infected cells demonstrated a 100-fold decrease in RNA concentration. This confirms that EILV-specific non-structural proteins fail to support significant levels of transcription and replication in NIH 3T3 cells.

Analysis of the composition of viral particles produced by the chimeric viruses revealed an important new feature: in mosquito cells, these viruses package both genomic and SG RNAs (**Fig 1C and 1D**). Both RT-qPCR and electrophoretic analysis of metabolically [^3^H]uridine-labeled RNAs demonstrated large amounts of SG RNAs in the viral particles, which were harvested from C7/10 cells well before the development of noticeable cytopathic effects (CPE) and further purified by ultracentrifugation through 25% sucrose. This was the result either of the inefficiency of the VEEV capsid protein binding to the heterologous EILV-specific RNA packaging signal (PS) [31] or as described for Aura virus [32], a natural ability of EILV to nonspecifically package the abundant SG RNAs. Regardless, the released virions could be potentially exploited as delivery vehicles for both genomic and SG RNAs. Indeed, infection of NIH 3T3 and other vertebrate cells with mosquito cell-derived, chimeric EIL/GFP/VEEV viruses resulted in reporter GFP gene or VEEV TC-83 structural protein expression despite an absence of viral RNA replication or *de novo* transcription of the SG RNA (**S2 Fig**).

**Fig 2:**
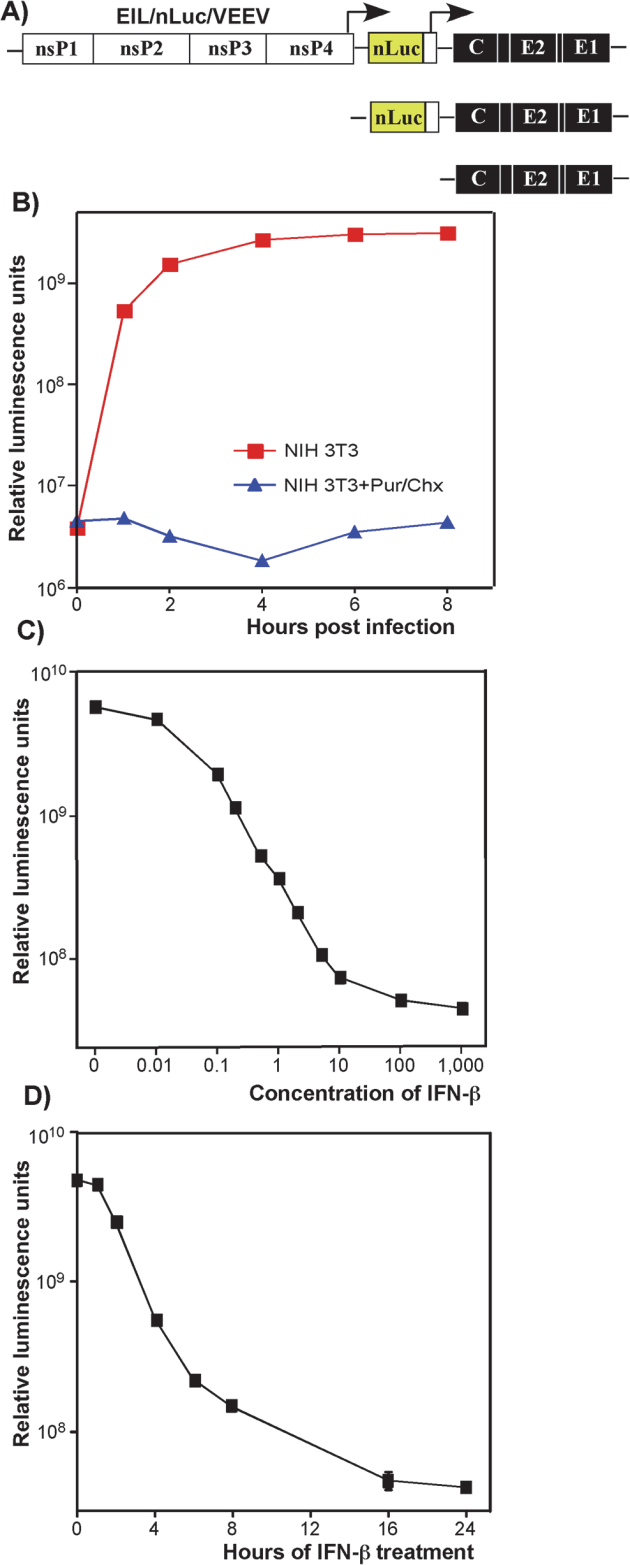
Type I IFN pretreatment inhibits nLuc expression in vertebrate cells in time- and concentration-dependent mode. **(A)** The schematic representation of EIL/nLuc/VEEV genome and encoded SG RNAs. **(B)** NIH 3T3 cells were infected with purified EIL/nLuc/VEEV at an MOI of 10 PFU/cell. After 1 h incubation at 4°C, cells were washed with cold PBS, and then incubated at 37°C in complete media. At the indicated time points, cells were harvested, and nLuc activity was assessed. The control cells were infected and further incubated in the presence of 50 μg/ml puromycin (Pur) and 50 μg/ml cycloheximide (Chx). **(C)** NIH 3T3 cells were treated for 20 h with IFN-β at the indicated concentrations. Cells were then infected with EIL/nLuc/VEEV at an MOI of 10 PFU/cell for 1 h at 37°C, washed with PBS, and incubated in complete medium at 37°C. nLuc activity was assessed at 4 h post infection. **(D)** NIH 3T3 cells were treated for indicated times with IFN-β at a concentration of 100 IU/ml. Cells were then infected with EIL/nLuc/VEEV at an MOI of 10 PFU/cell, washed with PBS, and incubated in complete media at 37°C. nLuc activity was analyzed at 4 h post infection. All results are presented as average of triplicate +/- SD in relative luminescence units from a single representative experiment. Most of standard deviations are too small to be seen. The experiments were reproducibly repeated three independent times.

To analyze expression of another reporter, nLuc, from the delivered SG RNA, mosquito cell-derived EIL/nLuc/VEEV chimeric virus was purified to homogeneity by continuous sucrose gradient ultracentrifugation. Then, NIH 3T3 cells were infected at 4^°^C and further incubated at 37^°^C. nLuc was expressed efficiently within the first 4 h post infection, but after this, an increase in Luc activity was no longer detected (**Fig 2A and 2B**), which suggested a lack of *de novo* nLuc RNA synthesis. Luciferase expression was not detected in the infected cells treated with the protein synthesis inhibitors puromycin and cycloheximide. In contrast, EIL/nLuc/VEEV-infected C7/10 cells demonstrated a continuous increase in nLuc expression for at least 24 h (**S3 Fig**), reflecting the reporter SG RNA synthesis.

The lack of RNA replication of EIL/VEEV-based viruses in vertebrate cells makes them of little value for studying alphavirus pathogenesis and virus-host interactions. However, these viruses represent a unique experimental tool, which allows designing and packaging into infectious virions a very wide variety of mRNA (SG RNAs), and studying the effect of IFN pretreatment on the early steps of alphavirus infection in a variety of experimental conditions in the absence of viral RNA synthesis/replication.

### Type I IFN treatment inhibits translation of alphavirus-specific RNAs

Expression of heterologous genes encoded by SG RNAs of chimeric viruses was sensitive to IFN-β pretreatment. The pre-treated cells produced no GFP (**S2 Fig**) or nLuc (see below) upon infection with EIL/GFP/VEEV and EIL/nLuc/VEEV, respectively. The inhibition of nLuc expression depended on both concentration of IFN-β and the time of pretreatment relative to infection (**Fig 2C and 2D**). Pretreatment with IFN-β at a concentration of 1 IU/ml for 20 h inhibited nLuc expression by 10-fold, and concentrations above 100 IU/ml reduced it to background levels (**Fig 2C**). Four-hour-long incubation of cells with IFN-β at a concentration of 100 IU/ml (**Fig 2D**), also decreased nLuc expression by 10-fold, and no expression above the background level was detected after 16 h of cell pre-incubation with IFN-β. In these experiments, the noticeably high background levels of nLuc activity were the result of packaging of nLuc protein into viral particles in C7/10 cells and then delivery together with viral RNAs (see the next section).

The experiments with EIL/VEEV-based RNA delivery demonstrated strong quantitative differences with data derived from the RNA transfection experiments [25]. Type I IFN pretreatment inhibited expression of the RNA-encoded genes not within 10 fold, but by more than two orders of magnitude. However, regulation of expression of mRNAs delivered by viral particles may be not exclusively determined by translation. The early events in alphavirus infection, such as binding to cell surface receptors, endocytosis, fusion with the endosomal membrane, and intracellular transport and the release of nucleocapsid into the cytoplasm followed by its disassembly can all affect viral gene expression. Moreover, VEEV glycoproteins reportedly contribute to the development of virus resistance to type I IFN [33]. Thus, possible effect(s) of type I IFN treatment on other early steps of EIL/VEEV infection had to be investigated.

**Fig 3:**
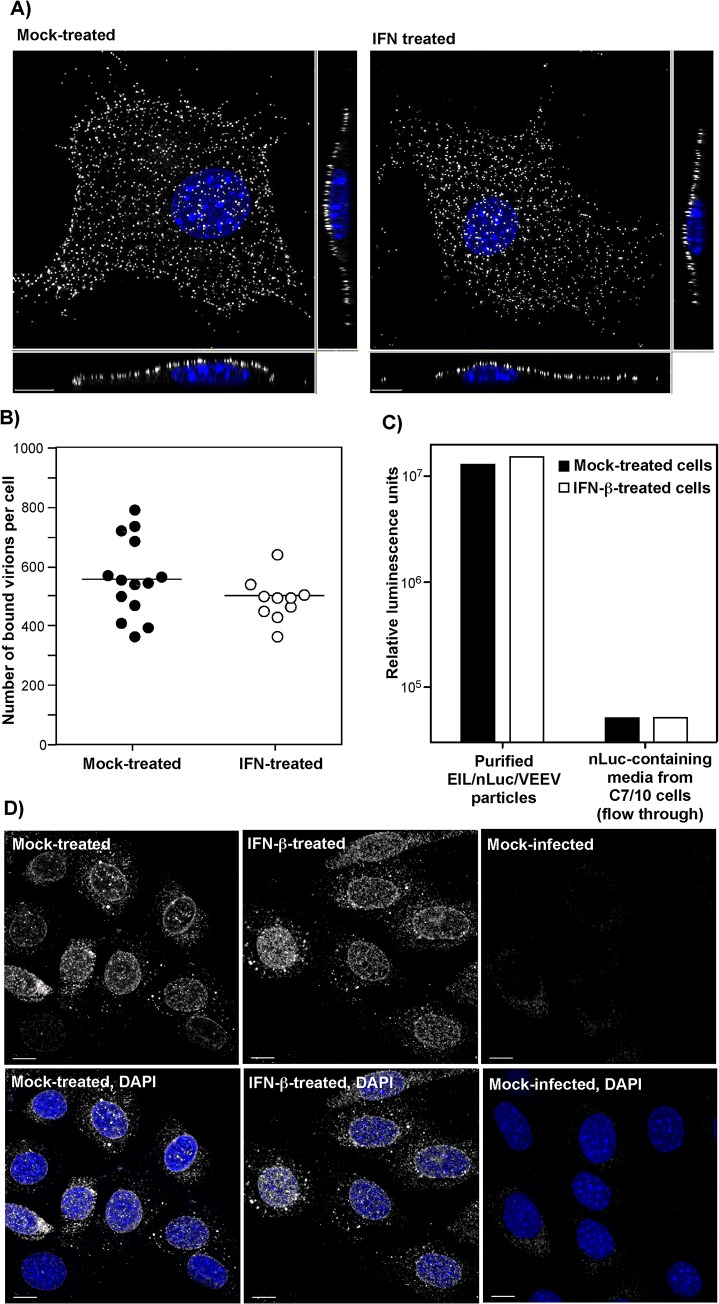
IFN-β treatment does not affect virus attachment, entry, or disassembly. **(A)** NIH 3T3 cells in Ibidi 8-well plates were treated for 20 h with IFN-β at a concentration of 100 IU/ml or mock-treated. Then cells were incubated with VEEV TC-83 (1.5x10^4^ PFU/cell) at 4°C for 1 hour, washed twice with cold PBS and fixed with PFA. Immunostaining of the adsorbed viral particles was performed immediately without any treatment with nonionic detergent using VEEV-specific mouse Abs and AlexaFluor555-labeled secondary Abs. Cell nuclei were stained with Hoechst dye. The three-dimensional images were acquired by confocal microscopy (see Materials and Methods for details), and **(B)** numbers of bound viral particles per cell were determined using Spot function of the Imaris software. **(C)** IFN-β-treated or non-treated NIH 3T3 cells were incubated with purified EIL/nLuc/VEEV (MOI of 20 PFU/cell) at 4°C for 1 hour, washed with cold PBS, lysed in Triton X-100-containing buffer, and nLuc activity in the samples was determined. Media from EIL/nLuc/VEEV-infected C710 cells was filtered using 100 kD centrifugal filters to eliminate viral particles and used as a flow-through, virus-free nLuc-containing control. **(D)** NIH 3T3 cells were treated overnight with 100 IU/ml of IFN-β or left mock-treated. They were incubated with concentrated virus (7.5x10^3^ PFU/cell) at 4°C for 1 h to allow virus interaction with cell surface receptors. Then incubation continued at 37°C for 1 h in complete media in the presence of cycloheximide. Cells were fixed with PFA, permeabilized and stained using rat mAb specific to the amino-terminal fragment of VEEV capsid, and AlexaFluor555-conjugated secondary antibody. Nuclei were stained with Hoechst dye. Images are presented as multiple intensity projections of 6 optical sections. Scale bar is 10 μm.

The potential effects of type I IFN pretreatment on virus attachment to plasma membrane were studied by two different approaches. First, we infected NIH 3T3 cells, pre-treated with IFN-β, and mock-treated, with equal numbers of PFUs of VEEV virions at 4^°^C for 1 h. Then cells were fixed and immunostained using VEEV-specific Abs without permeabilization of the plasma membrane. The three-dimensional (3D) images of multiple randomly selected cells were acquired on a confocal microscope (**Fig 3A**) and used to quantify the numbers of cell-bound virions (**Fig 3B**) (see Materials and Methods for details). No statistically significant difference (*P* > 0.1) was detected between the IFN-β- and mock-treated cells (**Fig 3B**).

The second approach took advantage of the ability of VEEV and other alphavirus virions to nonspecifically package detectable levels of other cytoplasmic proteins and even larger protein complexes, such as ribosomes [34], into released infectious virions. Virions released from EIL/nLuc/VEEV-infected C7/10 cells were purified to homogeneity by density gradient ultracentrifugation. The residual nLuc in these purified samples co-pelleted with viral particles, with no Luc activity remaining in the supernatants, strongly suggesting that nLuc was associated with purified virions. Then, equal amounts of the purified EIL/nLuc/VEEV virions were adsorbed to IFN-β- and mock-treated NIH 3T3 cells at 4^°^C, and after extensive washing with PBS, cells were lysed and assessed for nLuc activity. No measurable difference was found between the samples (**Fig 3C**). To avoid reaching saturation conditions in the above experiments, the amount of nLuc-containing virus used for binding was optimized to remain in the linear range (**S4 Fig**). As a control, we used soluble nLuc-containing, flow-through media, which passed centrifugal filters used for virus concentration. Despite this fraction having a few orders of magnitude higher original nLuc concentration than the fraction of purified viral particles, the amount of cell-binding nLuc in these media samples was 500-fold lower (**Fig 3C**).

For analysis of virus entry and disassembly, NIH 3T3 cells were treated with 100 IU/ml of IFN-β for 20 h or mock-treated. Cells then were infected with EIL/nLuc/VEEV at 4^°^C, and further incubated for 1 h at 37^°^C in the presence of cycloheximide to allow virus entry and disassembly, but not translation of the proteins from the delivered RNAs. Staining of fixed and permeabilized cells with a VEEV capsid-specific mAb demonstrated that in both IFN-β- and mock-treated cells, capsid was present in the cytoplasm (**Fig 3D**) with characteristic accumulation in the nuclear pores [35]. Its distribution and concentrations were indistinguishable in IFN-β- and mock-treated cells. The mAb used was specific to the very amino terminal fragment of capsid and did not interact with assembled nucleocapsids. Thus, the detected staining reflected nucleocapsid disassembly.

Taken together, these results strongly suggested that IFN-β treatment does not affect virus entry or disassembly, and thus, inhibition of translation of the incoming mRNAs appears to be a dominant mechanism regulated by IFN-β. These results are generally consistent with the data from RNA transfection-based experiments [25], but strongly underscore the effect of IFN-β pretreatment on translation of alphavirus-specific RNAs.

### IFN-β pretreatment differentially affects translation of mRNAs, containing 5'UTRs derived from different alphavirus genomes

**Fig 4:**
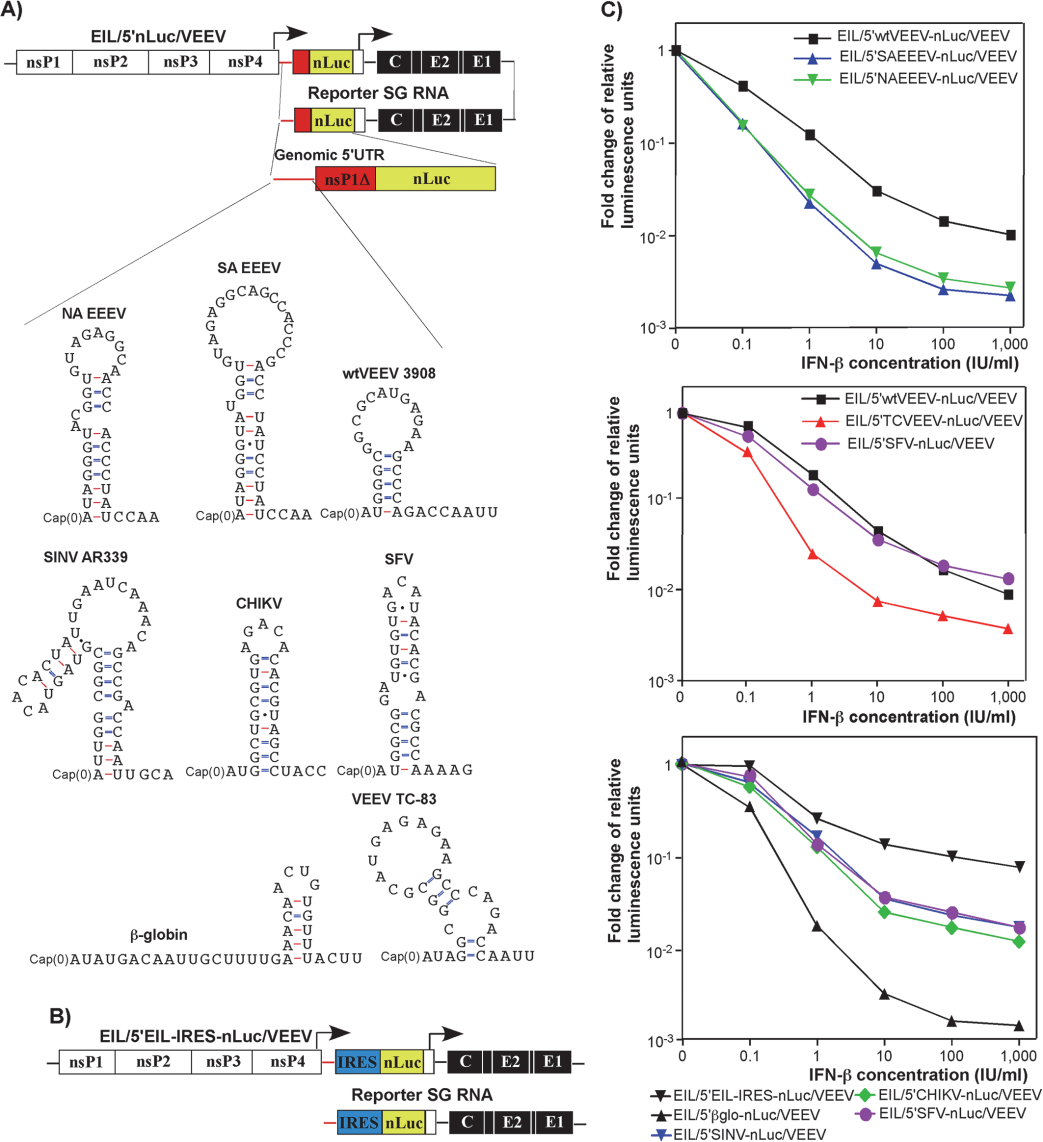
Levels of resistance of alphavirus RNA translation to IFN-β is determined by 5’UTRs of their genomes. **(A)** The schematic representation of EIL/5’nLuc/VEEV genomes with different 5’UTRs in the nLuc-encoding SG RNAs and the computer predictions (m-Fold) of the RNA foldings. **(B)** The schematic representation of the EIL/5’EIL-IRES-nLuc/VEEV genome, containing EMCV IRES downstream of the EILV SG RNA-specific 5’UTR. Because of the IRES presence, nLuc is likely translated from both viral genome and SG RNA. **(C)** wt MEFs were treated for 20 h with different concentrations of IFN-β or remained mock-treated and then infected with the indicated viruses for 1 h at 37°C. The MOI used was adjusted for each virus to obtain similar nLuc activity in the mock-treated samples. After infection, cells were incubated in complete medium at 37°C, and nLuc activity was assessed at 4 h post infection. The results were normalized to the nLuc activity in the samples of mock-treated, infected cells. The experiments were repeated multiple times with reproducible results. Panels represent the data obtained in a single experiment. All of the measurements were performed in triplicates, and standard deviations are too small to be seen.

The additional nLuc SG RNA in the EIL/nLuc/VEEV genome is dispensable for virus replication, and thus, such reporter SG RNA can be modified without affecting synthesis of proteins involved in viral genome replication and packaging in C7/10 cells. Accordingly, we modified the 5'-end of the nLuc-encoding reporter SG RNA to mimic the 5'UTRs of the genomes of different New and Old World alphaviruses (**Figs 4A and S5**). To preserve characteristic stem-loop structures that are predicted in the 5'UTRs, we used computer m-Fold predictions and cloned longer (~200 nt) fragments upstream of the nLuc ORF. These fragments contained the natural initiating AUG and encoded the amino terminal fragments of the corresponding nsP1 proteins (nsP1Δ), fused in frame with nLuc (**Fig 4A**). The additional engineered control constructs included those containing a 5’UTR of β-globin mRNA (EIL/5'βglo-nLuc/VEEV), which was additionally modified to lack a stable stem-loop structure, and EMCV IRES (EIL/5'EIL-IRES-nLuc/VEEV) (**Fig 4B**). The latter sequence was cloned between the EILV SG RNA-specific 5’UTR and nLuc to drive cap-independent translation.

All of the recombinant viruses were rescued from the constructs after RNA transfection into C7/10 cells. As described above (Fig 1C and 1D), the released viral particles contained high concentrations of nLuc-encoding reporter SG RNA with engineered 5’UTRs. Next, murine fibroblast cells either were treated with different concentrations of IFN-β or mock-treated, and then infected at the same MOI with the designed recombinant viruses. Translation of the delivered reporter SG RNAs was evaluated at 4 h post infection. This experimental format provided the most robust comparison of the effect of type I IFN pretreatment on translation efficiency. The results were highly reproducible and some are presented in **Fig 4C**. The following patterns were observed:

1. Among the New World alphaviruses, the 5’UTR of the epizootic strain of VEEV 3908 made translation of the nLuc template more resistant to IFN-β pretreatment than mRNAs containing the 5’UTRs of North and South American eastern equine encephalitis virus strains (NA EEEV and SA EEEV) (**Fig 4C**, *top panel*). This is consistent with EEEV having a lesser need to resist IFN, which it induces poorly during early stages of infection [36].
2. Translation of nLuc downstream of the 5'UTR of the vaccine strain of VEEV TC-83 was inhibited to a greater degree by IFN-β even though VEEV TC-83-specific 5’ terminus differs from VEEV 3908 by only a single nucleotide (G3A mutation) (**Fig 4C**, *middle panel*). These results were consistent with prior studies showing a greater IFN sensitivity of the attenuated VEEV TC-83 compared to its virulent parent strain, Trinidad Donkey [33, 37].
3. The 5'UTR of wt SINV (AR339), Semliki Forest virus (SFV), and CHIKV promoted translation of nLuc after pretreatment with IFN-β with a comparable efficiency to that of VEEV 3908-specific 5'UTR (**Fig 4C**, *middle and bottom panels*).
4. Translation of nLuc downstream of the modified β-globin-specific 5'UTR was the most sensitive to IFN-β pretreatment.
5. Pre-incubation of cells with IFN-β had the least effect on IRES-dependent translation of nLuc. However, a concentration of IFN-β above 10 IU/ml reproducibly decreased the efficiency of IRES-mediated translation.

Collectively, the data suggest that the 5'UTRs of alphaviruses vary in their ability to promote translation in the presence of ISGs induced by IFN-β. However, we cannot rule out an unlikely possibility that the applied IFN-β pretreatment might also differentially affect stability of delivered SG RNAs with different 5’ termini.

### IFIT1 has a critical role in IFN-induced inhibition of translation of alphavirus-specific RNAs

**Fig 5:**
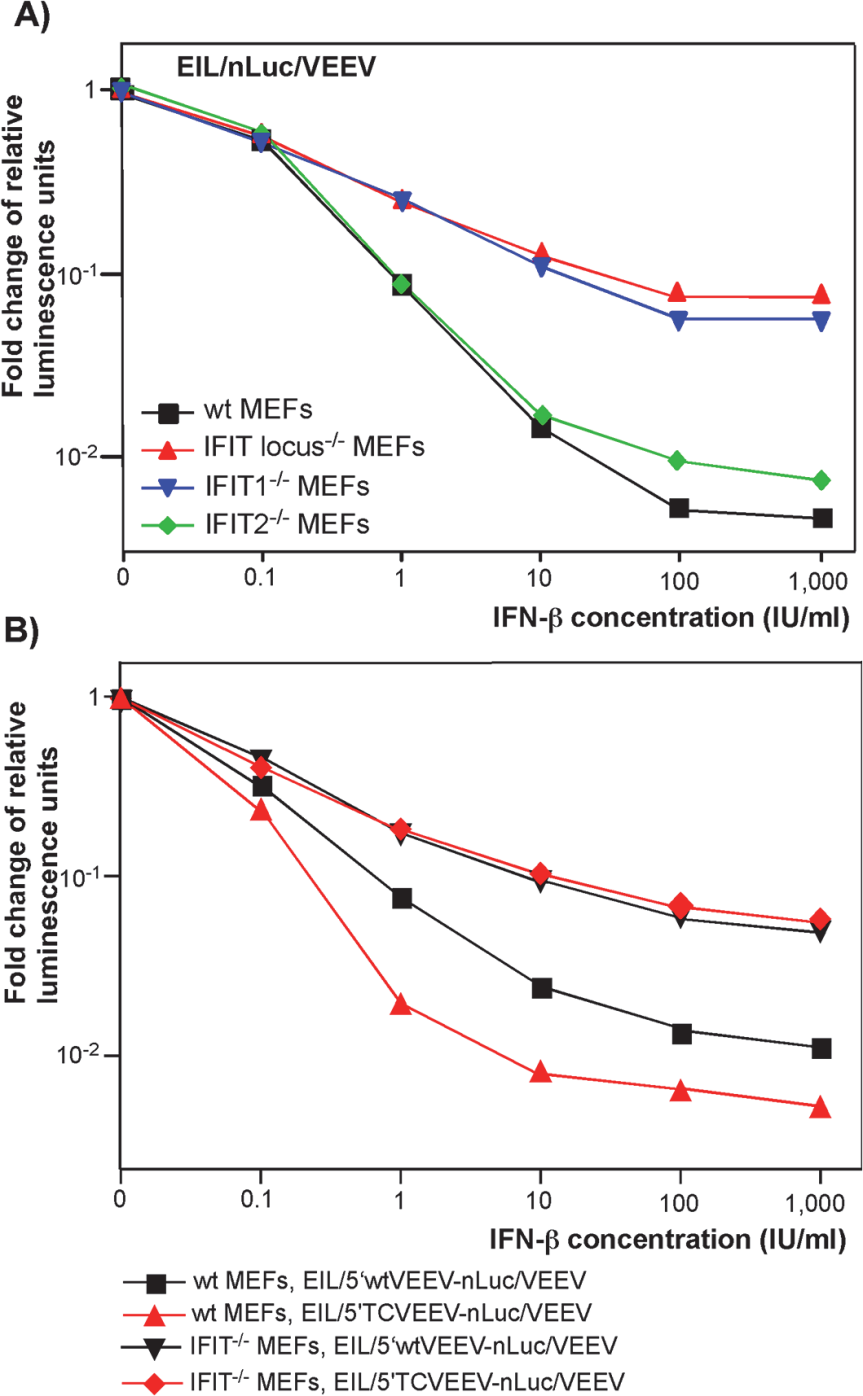
IFIT1 inhibits translation of incoming alphavirus-specific RNAs. **(A)** Wild type (wt), IFIT1^-/-^, IFIT2^-/-^ or IFIT locus^-/-^ MEF were treated for 20 h with the indicated concentrations of IFN-β or remained mock-treated. Then cells were infected with EIL/nLuc/VEEV at an MOI of 10 PFU/cell for 1 h at 37°C, washed with PBS, and further incubated in complete medium at 37°C. nLuc activity was analyzed at 4 h post infection, and data were normalized to samples from the mock-treated, infected cells. **(B)** Wt and IFIT1^-/-^ MEF were treated for 20 h with the indicated concentrations of IFN-β or remained mock-treated. Then cells were infected with EIL/5’wtVEEV-nLuc/VEEV and EIL/5’TCVEEV-nLuc/VEEV viruses. nLuc activity was analyzed at 4 h post infection and normalized to the activity in the samples of the mock-treated, infected cells. One of the reproducible repeated experiments is presented. All of the measurements were performed in triplicates, and standard deviations are too small to be seen.

The features that distinguish alphavirus genomic and SG RNAs from cellular mRNAs are the absence of *2'-O* methylation of the penultimate nucleotide of the cap [cap(0)] [38] and the presence of a stable stem-loop secondary structure at the 5' termini of viral genomic and SG RNAs [1]. As prior studies reported that IFIT1 functions as a key inhibitor of translation of cap(0)-containing mRNAs [39], we experimentally tested the roles of different IFIT protein family members in the development of the type I IFN-induced translation inhibition of alphavirus-specific RNAs. Murine embryonic fibroblasts (MEFs) derived from wt, *IFIT1* ^-/-^, *IFIT2* ^-/-^, or *IFIT* locus^-/-^ mice were treated with different concentrations of IFN-β for 20 h and infected with EIL/nLuc/VEEV. The activity of nLuc was assessed at 4 h post infection and normalized to that detected in mock-treated cells. Compared to wt MEFs, the IFIT1^-/-^counterparts demonstrated markedly less inhibition of translation of the delivered SG nLuc RNA in response to IFN-β pretreatment (**Fig 5A**). The deletion of the entire locus (IFIT1, IFIT2, and IFIT3) did not cause much additional change: IFIT locus^-/-^ and IFIT1^-/-^ MEFs exhibited essentially the same downregulation of nLuc translation. IFIT2^-/-^ MEFs demonstrated an IFN-β-dependent decrease in nLuc expression similar to that detected in wt MEFs (**Fig 5A**). Thus, the experiments suggest little if any antiviral role for IFIT2 and IFIT3 in this context, despite IFIT2 was readily detectable in the wt and IFIT1^-/-^ MEFs by Western blot, and its expression was strongly induced by IFN-β treatment. Importantly, in contrast to wt MEFs, the IFIT1^-/-^ MEFs did not discriminate between the wt VEEV 3908- and attenuated VEEV TC-83-derived 5’UTRs (**Fig 5B**). After IFN-β pretreatment, inhibition of translation of the SG RNAs containing either 5’UTR was equally inefficient.

Thus, IFIT1 appears to be the primary regulator of translation of the incoming viral RNA among IFIT family members. In the used experimental system, other IFITs did not play detectable role in translation regulation. However, other IFN-inducible factors, which remain to be characterized, account for an additional, 10-fold or more inhibition of translation of alphavirus-specific RNAs upon their natural delivery in viral particles (Fig 5). They need to be identified and further characterized.

### Canonical and noncanonical pathways in IFIT1 induction

**Fig 6:**
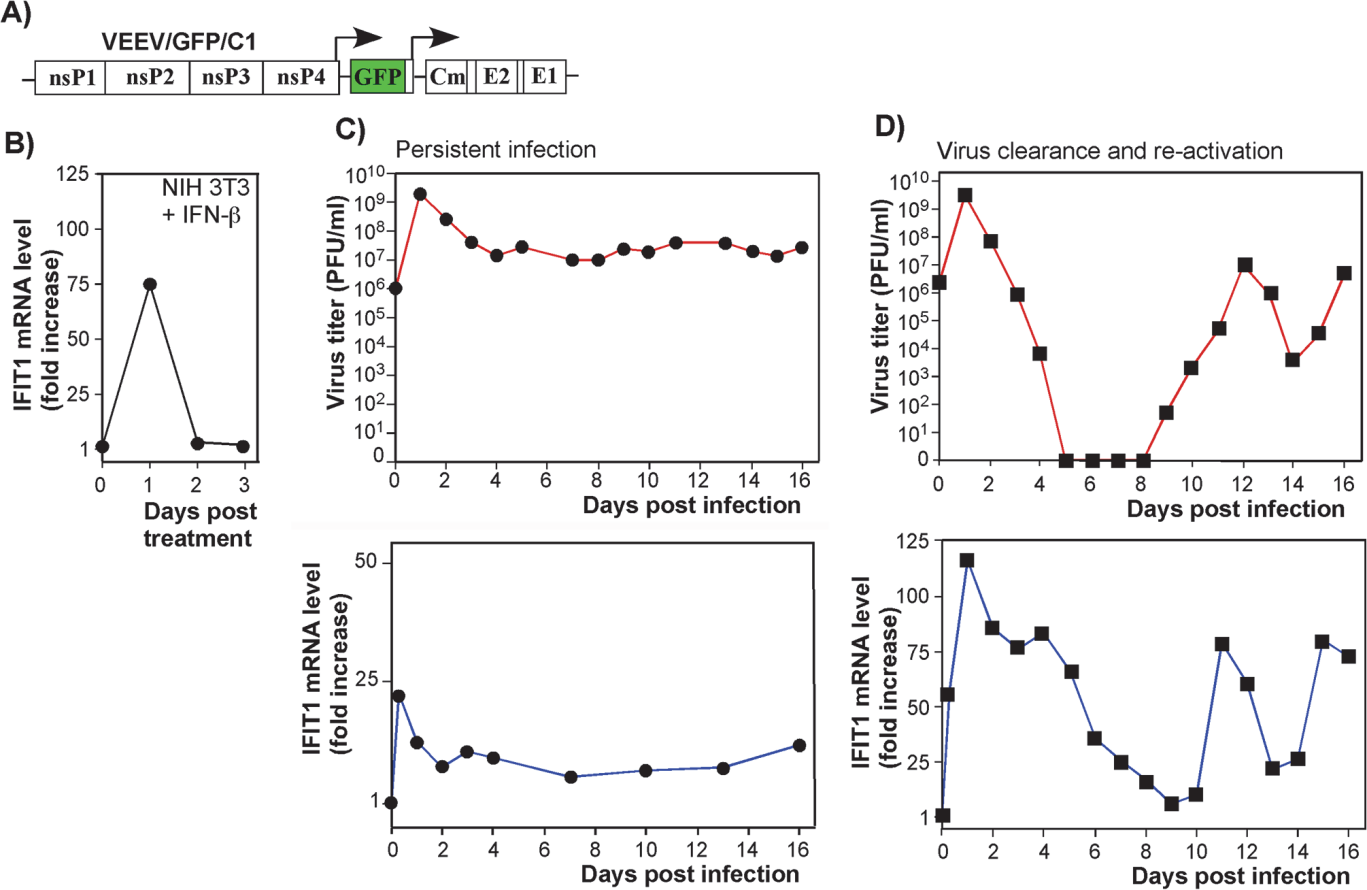
IFIT1 is expressed via IFN-dependent and alphavirus replication-dependent pathways. **(A)** The schematic representation of VEEV TC-83-based recombinant virus genome (VEEV/GFP/C1) containing mutations in the capsid-specific NLS. **(B)** The profile of IFIT1 induction in the NIH 3T3 cells treated for 24 h with IFN-β (500 IU/ml) and then incubated in IFN-free media. **(C)** IFN-α/βR^-/-^ MEFs were infected with VEEV/GFP/C1 at an MOI of 20 PFU/cell. Media were replaced at the indicated time points, and cells were harvested for RNA isolation and analysis. Panels represent titers of the virus and IFIT1 mRNA accumulation profiles. **(D)** NIH 3T3 cells were infected with VEEV/GFP/C1 at an MOI of 20 PFU/cell. As in the experiment presented in panel (C), media were replaced at the indicated times post infection, and RNAs were isolated from the cells. Panels represent titers of the virus and IFIT1 mRNA accumulation profiles. Two independent RNA samples were prepared for each time point for VEEV/GFP/C1-infected cells and three RNA samples for IFN-β-treated cells.

As IFIT1 is induced both by IFN-dependent (canonical) and IFN-independent (noncanonical, *e*.*g*., IRF3-dependent) mechanisms [40], we analyzed the contributions of these pathways to IFIT1 induction. Both components were clearly detected in microarray-based experiments [17] with IFN-β-treated and noncytopathic VEEV (VEEV/GFP/C1)-infected cells (**Fig 6**). The latter virus encodes a mutated capsid protein, which is incapable of inhibiting cellular transcription (**Fig 6A**) [12]. IFIT1 gene expression was strongly induced by the canonical, IFN-β-dependent, pathway: cells treated with IFN-β for 24 h demonstrated a ~75-fold increase in the presence of IFIT1-specific mRNA (**Fig 6B**). Within 24 h after the cessation of IFN-β-treatment, the levels of IFIT1-specific mRNA returned to baseline. The IFN-independent induction pathway was evident in IFN-α/βR^-/-^ MEFs, which lack the ability to respond to type I IFN treatment. In these cells, VEEV/GFP/C1 developed a persistent infection (**Fig 6C**, *top panel*), characterized by an increase in IFIT1 mRNA expression in the absence of type I IFN signaling (**Fig 6C**, *bottom panel*). During the persistent stage of virus replication, cells demonstrated 5-10-fold higher than baseline expression levels of IFIT1, which apparently played a role in replication control, but was insufficient for virus clearance. In comparison, wt cells infected with the mutant VEEV showed clearance at days 5–8 post infection (**Fig 6D**, *top panel*). This clearance correlated with higher levels of IFIT1 mRNA accumulation, which were even greater than those detected in IFN-β-treated cells (**Fig 6D**, *bottom panel*), indicating a synergistic or additive effects of virus replication and autocrine effects of type I IFN on induction of IFIT1. The previously described virus reactivation [12, 17], occurring at day 9–10 post infection (Fig 6D), was also accompanied with an increase in IFIT1 expression. This apparently contributed to the control of virus replication at lower levels.

### IFIT1 protein expression levels correlate with inhibition of alphavirus replication

**Fig 7:**
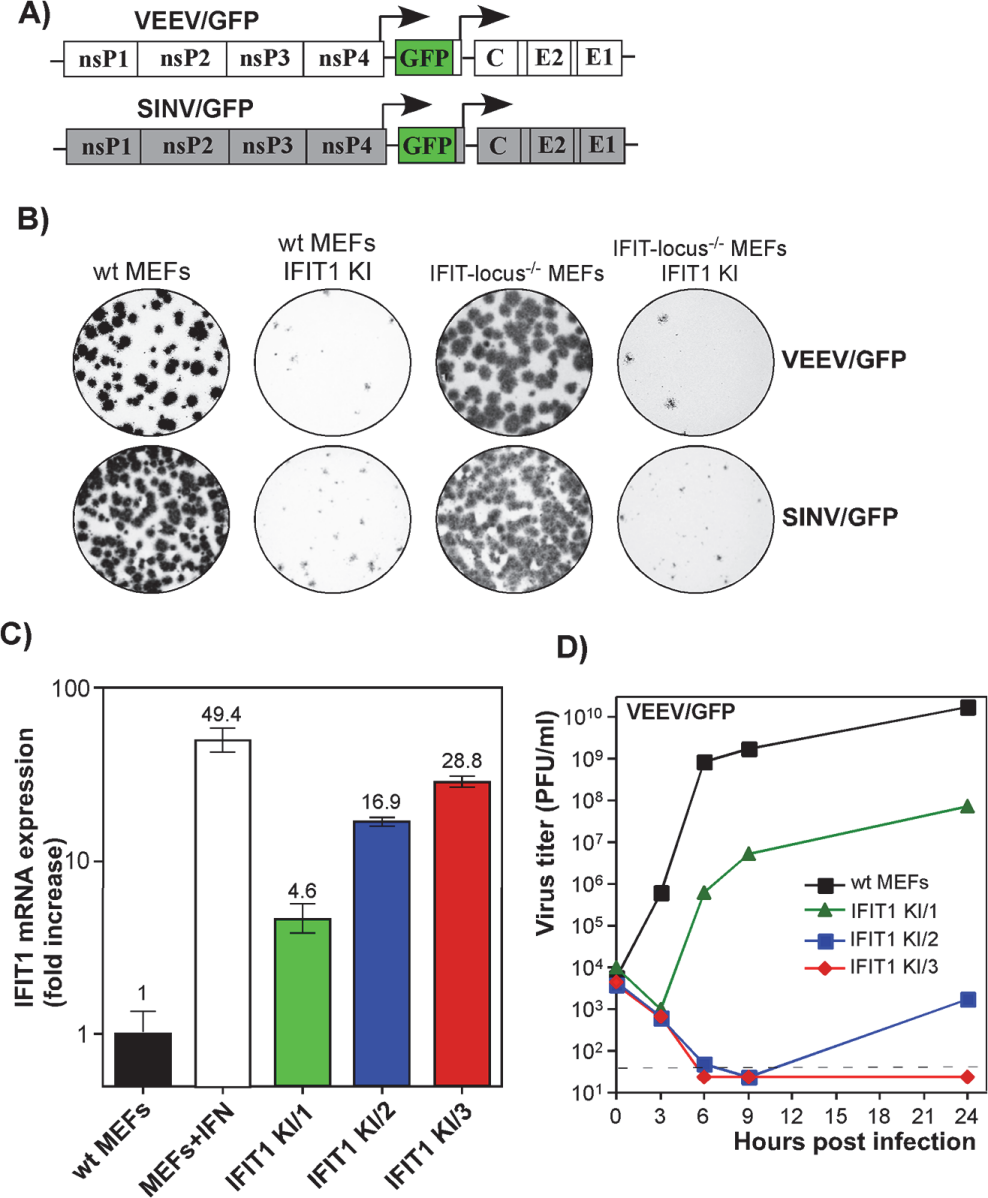
IFIT1 inhibits replication of alphaviruses in a concentration-dependent mode. **(A)** The schematic representation of the genomes of alphaviruses used in the experiments presented in this figure. **(B)** Populations of Bla^R^, IFIT1-expressing and parental cells were used for standard plaque assay of VEEV/GFP and SINV/GFP viruses. Since both viruses were unable to induce plaque formation on stable IFIT1-expressing cells, foci of GFP-expressing cells were detected on a Typhoon phosphorimager. **(C)** The results of RT-qPCR analysis of IFIT1-specific mRNA in different clones of IFIT1 KI cells, derived from wt MEFs, which were used in the experiments presented on the next panel and in the following sections. **(D)** The indicated clones of IFIT1 KI cells were infected at an MOI of 1 PFU/cell with VEEV/GFP. Media were replaced at the indicated time points, and virus titers were determined by plaque assay on BHK-21 cells.

Our data suggested that IFIT1 had a critical role in inhibiting alphavirus infection and promoting virus clearance from infected cells, and that the antiviral effect may be determined by the level of gene activation/IFIT1 protein expression (**Fig 6**). To further test this hypothesis, we generated IFIT1-expressing stable cell lines in wt, IFIT locus^-/-^, or IFIT1^-/-^ MEFs. Regardless of the cell type used, ectopic expression of IFIT1 negatively affected spread of both SINV/GFP and VEEV/GFP (**Fig 7A**), derived from attenuated SINV strain Toto1101 and VEEV TC-83, respectively, and the efficiency of formation of GFP-positive foci (**Fig 7B**). In all of the generated IFIT1-expressing bulk cell lines, individual cells demonstrated different levels of GFP expression upon infection with these viruses, additionally suggesting the dependence of virus replication on the level of IFIT1 expression. The selection of IFIT1 KI wt MEFs (IFIT1 KI MEFs) clones indeed revealed different levels of IFIT1-specific RNA and corresponding protein. For the next series of experiments, we used clones demonstrating IFIT1 expression levels that were similar or lower than that detected in MEFs treated with 100 IU/ml of murine IFN-β for 20 h (**Figs 7C and S6**). The levels of VEEV TC-83 replication in the cloned cell lines correlated directly with the efficiency of IFIT1 expression. Levels of IFIT1 expression similar to that in the IFN-treated cells completely blocked replication of VEEV TC-83 (**Fig 7D**). Clones with lower concentrations of IFIT1 supported virus replication, albeit the titers of released viruses were 3 to 7 orders of magnitude below those detected in wt MEFs.

### Alphaviruses are differentially sensitive to IFIT1

**Fig 8:**
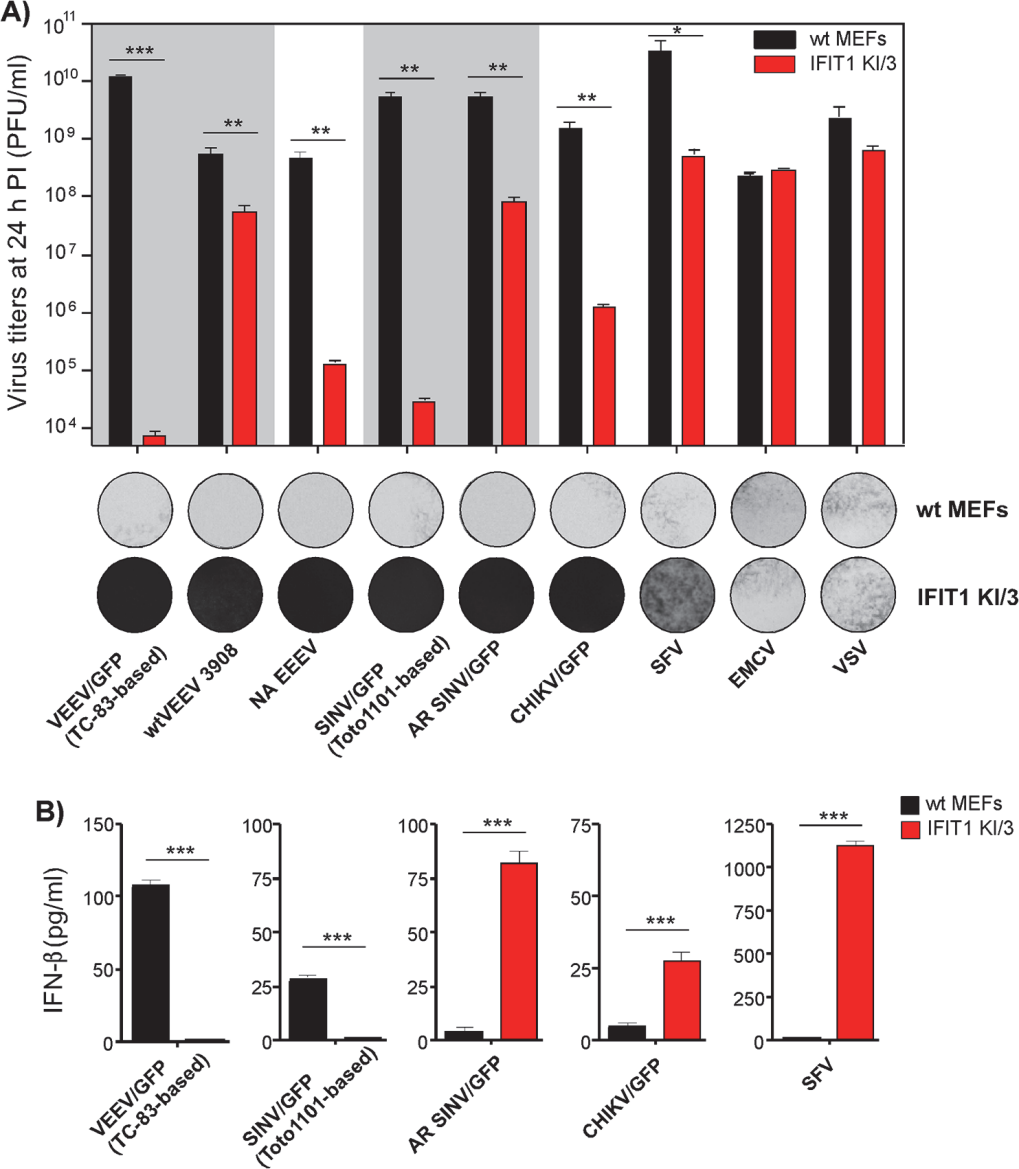
Wild type, but not tissue culture-adapted, alphaviruses are resistant to IFIT1-mediated replication inhibition and induce IFN-β in IFIT1-expressing cells. (**A**). Wt MEFs and IFIT1 KI/3 cells were infected with the indicated viruses at an MOI of 5 PFU/cell. Media were harvested at 24 h PI, and cells were stained with Crystal violet at 48 h PI. Virus titers were determined by plaque assay on BHK-21 cells. **(B)** Concentration of IFN-β was determined in the same harvested samples as described in the Materials and Methods. The experiments were performed two times and in triplicates. Statistical analyses were performed using unpaired T-test. *:p<0.05, **:p<0.01, ***:p<0.001. Means and SD of three experiments are presented

Next, we tested whether the differences in translation of SG RNAs containing 5’UTRs derived from different alphavirus genomes (**Fig 4**) correlate with resistance of virus replication to IFIT1 expression. The IFIT1 KI/3 cell line (IFIT1 ectopically expressed in wt MEFs), which demonstrated the highest resistance to TC-83-based VEEV/GFP (**Fig 7C and 7D**), and parental wt MEFs were infected with a panel of alphaviruses, which included wtVEEV 3908, VEEV/GFP (VEEV TC-83 vaccine strain-based virus), NA EEEV, SINV/GFP (Toto1101 laboratory strain-based virus), AR SINV/GFP (SINV/GFP with 5’UTR derived from natural isolate of SINV AR339), SFV, CHIKV/GFP and unrelated viruses (EMCV and VSV) at the same MOI. Titers of the released viruses were determined at 24 h post infection, and CPE was evaluated (**Fig 8A**). Consistent with the translation experiments, among the wt alphavirus isolates, NA EEEV replicated poorly in MEFs that ectopically expressed IFIT1. Other alphaviruses with wt 5’UTRs (wtVEEV 3908, AR SINV/GFP, SFV and CHIKV/GFP) demonstrated greater inherent resistance to IFIT1 and higher levels of replication. SFV was the least inhibited by IFIT1 in terms of infectious virus release and CPE development in IFIT1 KI/3 cells. Importantly, tissue culture-adapted variants of VEEV (VEEV/GFP) and SINV (SINV/GFP), containing known point mutations in their 5'UTRs resulting from passaging of wt viruses in cultured cells, were sensitized to the antiviral effects of IFIT1. The control, unrelated viruses EMCV (picornavirus) and VSV (rhabdovirus) replicated equally well in parental and IFIT1 KI/3 MEFs, confirming that IFIT1 does not substantially inhibit viruses that utilize IRES-dependent translation or have cap 1 structures on their mRNA [41, 42], and that ectopic expression of IFIT1 does not non-specifically inhibit virus replication.

### Alphaviruses with wt 5’UTRs become potent inducers of IFN-β when high levels of IFIT1 are present

Even though some of the wt alphaviruses, particularly SFV, showed inherent resistance to IFIT1, they did not form plaques on IFIT1 KI/3 MEFs (**Fig 8A)**. This decreased ability to spread and cause cell death suggested a possible second cell defense mechanism associated with IFIT1 expression. Indeed, IFIT1 KI/3 MEFs released high levels of IFN-β upon infection with viruses having wt 5’UTRs (**Fig 8B**). Remarkably, the amounts of IFN-β induced by these viruses were far greater in IFIT1-expressing cells than in wt MEFs despite the orders of magnitude lower yield of virus release. Viruses having 5’UTRs derived from tissue culture-adapted variants VEEV TC-83 and SINV Toto1101, demonstrated the opposite pattern of IFN-β induction: low, but detectable, levels of IFN-β in wt MEFs and undetectable levels of IFN release in IFIT1 KI/3 MEFs.

Some of the variants with wt 5’UTRs (AR SINV/GFP and CHIKV/GFP) were designed to express GFP from a second SG promoter. In wt MEFs, they produced GFP at levels readily detectable by fluorescence microscopy within 2 to 3 h post infection. However in IFIT1 KI/3 MEFs, the production of GFP was delayed by 8–10 h, suggesting markedly lower levels of virus replication. Thus, one explanation for the higher levels of IFN-β detected in IFIT1 KI/3 MEFs is that lower levels of virus replication in IFIT1-expressing cells, compared to wt MEFs, renders wt viruses less capable of interfering with cellular transcription and induction of IFN-β. Consequently, in the plaque assay the released type I IFN acted to protect uninfected cells from the slowly released viruses, which inhibited plaque formation.

### Alphavirus resistance to IFIT1 can be engineered genetically

**Fig 9:**
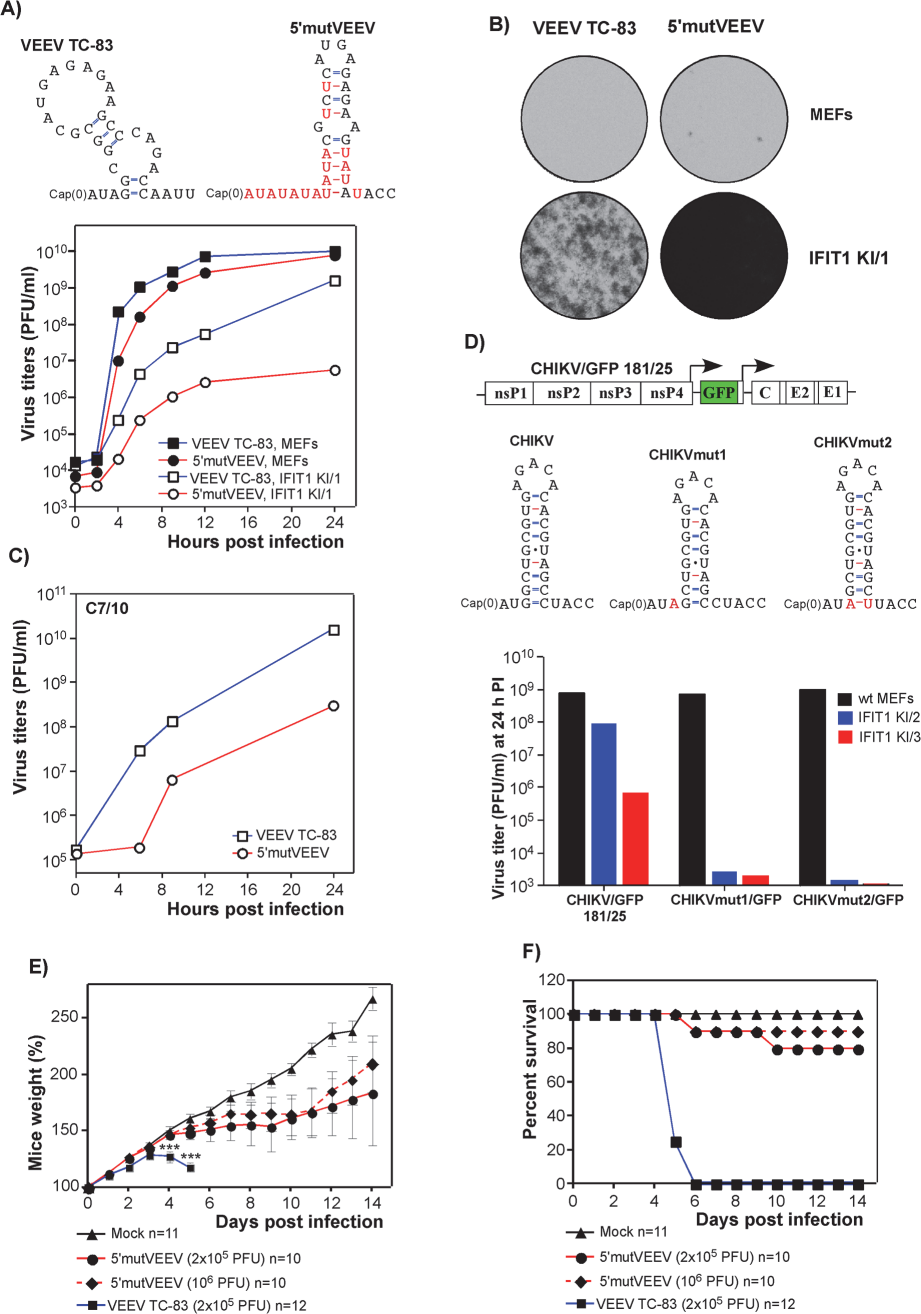
Alphavirus resistance to IFIT1 can be manipulated genetically and leads to virus attenuation. **(A)** The computer prediction of the 5’UTRs of the parental VEEV TC-83 and 5’mutVEEV viruses. To analyze the replication rates, wt MEFs and IFIT1 KI/1 cells were infected at an MOI of 5 PFU/cell with VEEV TC-83 and 5’mutVEEV, media were replaced at the indicated times PI, and virus titers were determined on BHK-21 cells. **(B)** At 48 h post infection, cells used for analysis of virus replication were stained with crystal violet to assess for CPE. **(C)** C7/10 cells were infected at an MOI of 10 PFU/cell with VEEV TC-83 and 5’mutVEEV, media were replaced at the indicated times PI, and virus titers were determined on BHK-21 cells. **(D)** The schematic representation of the CHIKV/GFP 181/25 genome and computer predictions of the original and newly designed 5’UTRs. Rescued viruses were used to infect wt MEFs, IFIT1 KI/2 and IFIT1 KI/3 cell lines at an MOI of 5 PFU/cell, and at 24 h post infection, virus titers were determined by plaque assay on BHK-21 cells. **(E and F)** Six-day-old NIH Swiss mice were infected via s.c. route with the indicated doses of VEEV TC-83 or the designed VEEV variant with mutated 5’UTR (5’mutVEEV). Mice were monitored daily in terms of weight change **(E)** and survival **(F)**.Unpaired T-test was used to compare weight change between the VEEV TC-83 and 5’mutVEEV groups (both 2x10^5^ and 10^6^ PFU) at day 4 and 5 post-infection. ***: p<0.001. Survival curves of the VEEV TC-83 and 5’mutVEEV groups (both 2x10^5^ and 10^6^ PFU) are significantly different (p-values <0.001).

The experiments evaluating the role of 5’UTRs on RNA translation (**Fig 4**) suggested that the VEEV TC-83-specific 5'-terminus makes the RNA more sensitive to type I IFN pretreatment than wt VEEV 5’UTR. However, the TC-83-specific 5'UTR is predicted to retain a considerable stem-loop structure (**Fig 9A**) that may weakly antagonize IFIT1, as the modified β-globin 5’UTR lacking secondary structure at the 5' end demonstrated even greater sensitivity to IFN-β pretreatment (**Fig 4C**). Based on these data and the results of our previous studies of the 5'-promoter structure and function [43–45], we designed an artificial 5’UTR in the VEEV TC-83 background (**Fig 9A**). This sequence was predicted (i) to remain functional as a promoter for RNA synthesis in mammalian cells due to the presence of multiple AU repeats, but (ii) to be a less efficient promoter in insect cells due to its lack of a 5'-terminal stem-loop structure, and (iii) to be more sensitive to IFIT1-specific inhibition because of its long 5'-terminal RNA fragment, which is not involved in base pairing [46]. The engineered construct 5’mutVEEV was viable and, based on infectious center assay, did not require additional adaptive mutations for replication. The rescued 5'mutVEEV replicated in wt MEFs with efficiency similar to that of the parental VEEV TC-83 (**Fig 9A**). However, in IFIT1 KI/1 MEFs expressing the lowest level of IFIT1, titers of released 5'mutVEEV were 20 to 200-fold lower than those of VEEV TC-83. Accordingly, in contrast to the parental virus, the designed mutant did not cause noticeable CPE in IFIT1 KI/1 cells (**Fig 9B**). An additional characteristic of this mutant, as predicted, was less efficient replication in mosquito cells (**Fig 9C**).

To extend these results, another set of 5'UTR mutants was designed based on the CHIKV/GFP (181/25 strain) virus. Since knowledge of the CHIKV 5’UTR promoter is less advanced compared to SINV and VEEV, we introduced only 1 or 2 point mutations (CHIKVmut1/GFP and CHIKVmut2/GFP, respectively), which destabilized the base of the predicted 5'-stem, but were not expected to affect the overall predicted secondary structure (**Fig 9D**). Both viruses were viable, and became sensitive to IFIT1. These CHIKV mutants replicated to the same level as parental virus in wt MEFs (**Fig 9C**), but did not replicate productively in either IFIT1 KI/2 or IFIT1 KI/3 MEFs: no GFP expression was detected, and virus titers remained at the level of detection, 3–5 orders of magnitude below the corresponding titers of parental CHIKV/GFP 181/25. Thus, a knowledge of both promoter structure and the antiviral function of IFIT1 enabled us to design increasingly attenuated variants of CHIKV and VEEV.

To confirm additional attenuation, we infected s.c. 6-day-old NIH Swiss mice with parent VEEV TC-83 and the 5'mutVEEV. All of the TC-83-infected mice failed to gain weight and succumbed to the infection within 6 days (**Fig 9E and 9F**). In comparison, only few of the mice infected with the same or higher doses of 5'mutVEEV died within 14 days of infection. 5'mutVEEV-infected mice still developed disease, which was detectable by their slower growth compared to those sham-infected, but most of them recovered.

## Discussion

To date, type I IFNs remain the most potent, broad-spectrum antiviral agents. IFN treatment of cells induces a large set of ISGs, which prevent infection with many viral pathogens, including alphaviruses. The list of currently known ISGs includes more than 300 members, but the exact mechanisms of function in inhibition of virus replication have been identified for only a small subset [18–24]. The detailed mechanistic investigation of the functions of individual ISGs is complicated by the difficulty of dissecting particular processes in virus replication independently of one another.

The recently discovered and characterized EILV alphavirus provided us with an opportunity to evaluate the effects of type I IFN pretreatment on the very early steps of the alphavirus replication cycle and to study them independently of viral RNA replication. Chimeras of EILV and VEEV cannot replicate in vertebrate cells, but in mosquito cells they package the engineered nLuc- or GFP-encoding SG RNAs into VEEV protein-encased particles, which then efficiently deliver them into vertebrate cells (**Fig 1**). Thus, such chimeras represent an efficient means of delivery of engineered SG RNAs into many cell types, and most importantly, such RNAs are delivered via the natural virus entry pathway. In these SG RNAs, 5’UTRs can be designed to completely mimic the 5'-ends of alphavirus genomes and then tested for promoting translation of reporter genes under different experimental conditions (**Fig 4**). Using this experimental system, we found that IFN-β pretreatment could block alphavirus infection before the beginning of RNA replication. IFN-β did not affect the steps of virus entry and virion disassembly (**Fig 3**), but as suggested previously [25], strongly inhibited translation of the incoming alphavirus RNA. The inhibitory effect depended on the time of IFN pretreatment and IFN-β concentration (**Fig 2**). The efficiency of translation of the engineered SG RNAs in the IFN-β-treated cells was also found to strongly depend on the origin of their 5’UTR (**Fig 4**). Translation of the RNAs containing 5’UTRs from the genomes of natural isolates of the Old World alphaviruses, SINV, CHIKV and SFV, in particular, was more IFN-resistant than from templates having non-alphaviral 5’UTRs. Among the New World alphaviruses, the 5’UTRs of NA or SA EEEVs were more sensitive to type I IFN, in terms of their ability to promote RNA translation, than 5’UTR derived from a wt epizootic VEEV genome. The previously described mutation of nt 3 in the VEEV TC-83 5’UTR made translation of this control template less resistant to IFN-β pretreatment, and this effect correlated with the previously described IFN-sensitive phenotype of the attenuated vaccine strain of VEEV [37]. The experimental data also suggested the existence of at least two mechanisms by which IFN-specifically inhibited translation of the alphavirus templates. One was determined by IFIT1 expression, and the second occurred through an IFIT1-independent pathway. The latter mechanism affected translation of the delivered capped RNAs in IFIT1^-/-^ MEFs, and downregulated translation of the EMCV IRES- rather than cap-containing templates in wt MEFs (**Figs 4 and 5**). It may be based on PKR/eIF2a- and/or PARP-mediated mechanism of translation regulation [22, 47].

IFIT1 is a member of a family of IFN-induced proteins with tetratricopeptide repeats [21]. Four IFIT family members have been characterized extensively in humans (*IFIT1* (also known as *ISG56*), *IFIT2 (ISG54*), *IFIT3* (*ISG60*) and *IFIT5* (*ISG58*)) and three members are expressed in mice: *Ifit1* (*Isg56*), *Ifit2* (*Isg54*), *Ifit3* (*Isg49*). IFIT1 has been described as a protein that binds viral RNA cap structures lacking *2'-O* methylation, which results in inhibition of binding of eukaryotic translation initiation factors and ultimately, downregulation of virus replication [41, 46, 48, 49]. Recent studies indicate that IFIT1 primarily interferes with the interaction of eIF4E with the cap structure [50]. Subsequently, it was shown that secondary structural motifs at the 5'-end of wt VEEV genomic RNA interfere more efficiently with IFIT1 binding than the mutation-containing 5'UTR of the attenuated VEEV TC-83 strain [39]. In our study, the previously identified difference in IFIT1 binding to wt and TC-83-specific 5’UTRs strongly correlated with IFN-β-induced translation inhibition of the RNA templates having the same 5'-termini. This difference in translation inhibition was no longer detectable in IFIT1^-/-^ MEFs (**Fig 5**), suggesting that type I IFN-induced expression of IFIT1 has a critical role in translational block of alphavirus infections and can discriminate between wt and mutated 5’UTRs. The results in IFIT1-transfected IFIT-locus^-/-^ MEFs (**Fig 7**) suggest that IFIT1 can function independently of other IFIT proteins to attenuate translation of alphavirus RNAs. Thus, other IFIT members have at best subordinate roles in inhibiting the translation of alphavirus RNAs. This inhibitory effect varies with the ability of IFIT1 to bind to RNA displaying 5'-ppp moieties, where a complex of IFIT1, IFIT2, and IFIT3 putatively is required [51]. IFIT1 expression in MEFs is induced by both the type I IFN-dependent pathway and replication of alphavirus-specific RNAs (**Fig 6**).

Resistance of translation of the mRNA templates containing 5’UTRs derived from different alphaviruses in IFN-β-treated cells correlated with the efficiency of replication of the corresponding viruses in the presence of higher levels of IFIT1 (**Figs 4 and 8**). CHIKV, SINV, SFV and VEEV with natural wt 5’UTRs replicated in IFIT1-expressing cell lines more efficiently than other alphaviruses (**Fig 8**). At a high MOI, SFV, the most resistant alphavirus, also induced detectable CPE. However, the direct interference with translation of alphavirus genomes appears to be not the only mechanism by which IFIT1 affects virus replication. Spread of IFIT1-resistant viruses with wild type 5’UTRs was inhibited by a second mechanism: in the presence of higher levels of IFIT1, these replicating viruses became potent inducers of type I IFN (**Fig 8**). In IFIT1-expressing cells, low titers of released viruses and very slow rates of accumulation of GFP indicated inefficient replication and expression of the encoded proteins. Capsid and nsP2 proteins of the New World and the Old World alphaviruses, respectively, function stoichiometrically to inhibit cellular transcription [35, 52]. Thus, in the presence of higher levels of IFIT1, the inefficiently replicating wt viruses likely were sensed by cellular pattern recognition receptors, such as RIG-I and MDA5. However, slow accumulation of viral proteins with transcription inhibitory functions (nsP2 or capsid) rendered them less capable of interfering with type I IFN activation. The released IFN-β activated the antiviral state in as yet uninfected cells and also probably had an autocrine effect on already established virus replication. Very inefficient replication of attenuated strains of VEEV (TC-83) and SINV (Toto1101) in the same IFIT1 KI/3 MEFs made the latter viruses, in turn, incapable of IFN-β induction at all (**Fig 8**).

Computer predictions and biochemical data indicate that a distinguishing characteristic of the 5’UTRs in alphavirus genomes is the presence of short stem-loop structures at the 5'-termini. A stem-loop structure at the 5'-end of VEEV has been confirmed by enzymatic analysis [53] and by nuclear magnetic resonance imaging [39, 53]. The nucleotide sequences and computer-predicted stem-loop structures differ among alphavirus species, but demonstrate a high degree of conservation between geographically distant isolates. Thus, as shown for VEEV and SINV [39], these secondary structures likely regulate the efficiency of interaction with IFIT1 with the 5'-terminal cap(0) and ultimately, the efficiency of translation of viral genomes in type I IFN-activated cells. Based on the accumulated experimental data, the molecular mechanism of this regulation of IFIT1-cap(0) interaction appears to be straightforward. To make alphaviruses capable of replication and IFIT1-resistant, the first two nucleotides need to be AU, and to be followed by a G-C-rich stem. Mutation of nt3, G to A, in the case of VEEV for example, or the presence of the wt AUA in both NA and SA EEEVs either makes first nucleotides unpaired (VEEV TC-83) or has strong negative effect on the stability of the hairpin base (EEEV). This in turn increases the efficiency of IFIT1 binding and reduces the viral resistance to IFIT1-mediated inhibition of translation. In contrast to other alphaviruses, EEEV is a poor inducer of type I IFN *in vivo* [10, 54]. Consequently, in the absence of type I IFN-induced IFIT1 induction, there is little selective pressure for EEEV to evolve an IFIT1 binding-resistant stem-loop in the 5’UTR.

The only alphavirus examined, which does not follow this rule, is SINV. Its 5'UTR starts with AUU, but the two uridine residues are predicted to form an RNA stem, with the GG nucleotides that follow downstream. This presence of U instead of more standard A in position 3 also might make binding of the first 3 nt to IFIT1 less efficient. Destabilization of following two G-C pairs by G5A mutation in Toto1101 affects the stability of the entire stem, promotes IFIT1-RNA interaction and makes SINV more sensitive to type I IFN [39] and the antiviral effects of IFIT1.

The existence of a simple means to reduce sensitivity to IFIT1 by increasing the stem stability raises the question as to why all alphaviruses have not evolved greater resistance to this ISG product. Indeed, we readily selected a VEEV mutant capable of more efficient replication in the presence of IFIT1. However, it should be noted that the IFIT1 protein is only one of many members of the highly redundant system of the ISG products. Its contribution to development of IFN-induced antiviral state is limited, and expression of other ISGs has deleterious effect on alphavirus replication; thus, selection of mutants with a maximal resistance level to IFIT1 appears not to be sufficiently beneficial for virus replication. There are also alternative explanations for why there is a limit to IFIT1 resistance in the 5' end. (A) Mutations in the 5'UTR that enhance the amount of double-stranded RNA character could sensitize viruses to other pathogen recognition receptors (e.g. RLR or TLR) or antiviral ISG products. (B) The 5'-terminal sequences in alphavirus genomes have other key functions including (i) facilitating translation of the nsPs, (ii) encoding promoter elements for synthesis of the negative strand of the viral genome, and (iii) their complementary sequence in the negative-strand RNA intermediate encodes promoters for synthesis of the positive strand [43–45, 53]. Thus, higher resistance to IFIT1, determined by the stability of stem-loop structures in the 5’UTRs, may be not beneficial for virus replication in both vertebrate hosts and/or mosquito vectors. The increase in resistance of alphaviruses to IFIT1 may have great cost in terms of reduction of promoter function and efficiency of viral replication. Importantly, the 5'-terminus of EILV genome is also predicted to form stable stem-loop (**S5 Fig**), but this virus does not replicate in vertebrates and thus, never interacts with IFIT1. However, the stem-loop structure of the EILV 5’UTR is similar to that of other alphaviruses, additionally suggesting its importance for promoter and perhaps other as yet undiscovered functions. One of them might be inhibition of cap(0) interaction with possible mosquito IFIT1 ortholog, which stays to be discovered.

Passaging of the alphaviruses in cultured cells, traditionally used to adapt them for more efficient replication, represents an interesting selection system. During serial passaging of alphaviruses, such as the 83 passages performed for development of the vaccine strain VEEV TC-83 [55, 56], resistance to IFIT1 no longer plays a significant role. As a result, VEEV TC-83 evolved an adaptive mutation in the 5’UTR [57, 58], which increased the rates of replication in cultured cells [53]. Thus, passaging led to enrichment of virus populations with variants adapted not only to binding to the heparan sulfate-containing moieties on the cell surface [59], but also to more efficient spread and growth to higher titers. This mutation in the 5’UTR, however, made VEEV less resistant to type I IFN [37]. Apparently, similar selection occurred in the process of development of the laboratory strain of SINV Toto1101 [60].

The accumulated and detailed knowledge about alphavirus promoter structures and functions [43–45, 53], and the new data from this and other studies regarding the resistance of alphaviruses to IFIT1, provide an opportunity to design new types of attenuated viruses (**Fig 9**). For instance, the engineered 5’mutVEEV replicated efficiently in mammalian, but not mosquito, cells and was more sensitive to the antiviral effects of IFIT1. This mutant virus was more attenuated than VEEV TC-83, which at least partially retains the secondary structure of the VEEV-specific 5’UTR. It also demonstrated higher level of attenuation in 6-day-old mice. Similarly, the re-engineered CHIKV 181/25 variants with mutated 5’UTR exhibited higher sensitivity to IFIT1-mediated inhibition of replication.

In summary, the results of this study demonstrate that (i) type I IFN pretreatment blocks translation of the alphavirus RNAs delivered in infectious virions, but not other early events in alphavirus infection. (ii) The IFN-induced inhibition of translation is determined by both IFIT1-dependent and IFIT1-independent mechanisms. (iii) Alphaviruses vary in their ability to replicate in the presence of IFIT1, and this difference is determined by the structures of the 5’UTRs in their genomes. (iv) The presence of IFIT1 at higher levels makes IFIT1-resistant wt alphaviruses more potent inducers of type I IFN. Thus, IFIT1 acts as both an antiviral effector molecule and inducer of innate immunity against alphaviruses. (v) Other members of IFIT family have subordinate roles in the inhibition of alphavirus replication. (vi) Our data provide a plausible explanation of the mechanism of alphavirus attenuation during their serial passaging in vaccine development. (vii) A mechanistic understanding of alphavirus 5’UTR promoter and immune evasion functions allows engineering of extensive, irreversible changes into 5’ termini of alphavirus genomes. These modifications affect virus replication in mosquito, but not mammalian cells, make such variants attenuated and can be used as one of the means in vaccine development.

## Materials and Methods

### Cell cultures

NIH 3T3 cells were obtained from the American Type Culture Collection (Manassas, VA). They were maintained in alpha minimum essential medium (αMEM) supplemented with 12.5% fetal bovine serum (FBS) and vitamins at 37°C. BHK-21 cells were provided by Paul Olivo (Washington University, St. Louis, MO) and were cultured in αMEM supplemented with 10% FBS and vitamins. Mosquito C7/10 cells were obtained from Henry Huang (Washington University, St. Louis, MO) and propagated at 30°C in Dulbecco’s modified Eagle’s medium (DMEM) supplemented with 10% heat-inactivated FBS and 10% tryptose phosphate broth (TPB). Wild type, IFIT1^-/-^, IFIT2^-/-^ and IFIT-locus^-/-^ murine embryonic fibroblasts (MEFs) were generated and maintained according to published protocols [61] in DMEM supplemented with 10% FBS. The IFIT-locus^-/-^ mice used to produce MEFs were generated by Ganes Sen (Cleveland Clinic, Cleveland OH), provided as a generous gift, and will be described elsewhere.

### Plasmid constructs

The original plasmids containing the infectious cDNAs of VEEV TC-83 (pVEEV and pVEEV/GFP), pVEEV 3908, SINV Toto1101 (pSINV), SINV/GFP Toto1101 (pSINV/GFP), CHIKV 181/25 (pCHIKV) and Eilat virus (pEILV) were described elsewhere [12, 30, 62–65]. Other plasmids were designed using standard PCR-based techniques. The introduced modifications are described in the Results. Sequences of the plasmids and details of the cloning procedures can be provided upon request.

### Rescuing of recombinant viruses

Plasmids containing the complete viral genomes were purified by CsCl gradient centrifugation and then linearized using the unique restriction sites located downstream of the poly(A) tails in the cDNA copies of viral genomes. The corresponding RNAs were synthesized in vitro using SP6 RNA polymerase in the presence of a cap analog as previously described [66]. The yield and integrity of the transcripts were analyzed by agarose gel electrophoresis under non-denaturing conditions. The transcription mixtures were used for transfection without further RNA purification. Viruses were rescued either by electroporation of the *in vitro*-synthesized RNAs into BHK-21 or C7/10 cells [44]. Viruses were harvested at 24 h post transfection of BHK-21 cells or at 48 h post transfection of C7/10 cells. Virus titers were determined using a standard plaque assay on C7/10 or BHK-21 cells [44, 67]. For some of the mutants, we assessed the infectivity of RNA by infectious center assay. In this assay, ten-fold dilutions of electroporated BHK-21 cells were seeded into 6-well Costar plates containing subconfluent, naïve BHK-21 cells. After 2 h of incubation at 37°C, media were replaced by 2 ml of MEM containing 0.5% of agarose and 3% FBS. Plaques were stained with crystal violet after 48 h of incubation at 37°C and infectivity was determined in PFU per μg of transfected RNA. The experiments with pathogenic alphaviruses were performed in the BSL3 facility of the UAB SEBLAB according to the IBC-approved protocols.

### Purification of chimeric viruses

Subconfluent C7/10 cells in 150-mm dishes were infected with EIL/VEEV or other chimeric viruses, containing nLuc or GFP genes with varying 5’UTRs under control of an additional SG promoter, at an MOI of 10 PFU/cell for 1 h at 30°C. Then cells were incubated overnight in complete media and then in serum-free VP-SF media (Invitrogen) for 24 to 30 h. These media were harvested before cytopathic effect (CPE) developed and additionally clarified by low-speed centrifugation. Viral particles were then concentrated using Amicon Ultra 100K centrifugal filters (Millipore) as described elsewhere [68]. In most experiments, viruses were used without further purification. In some experiments, concentrated samples were purified further by ultracentrifugation in continuous 20–50% sucrose gradients in a SW-40 rotor at 38,000 rpm for 3 h at 4°C. Visible bands of viral particles were collected, diluted in PBS, and concentrated by ultracentrifugation in discontinuous 25–50% sucrose gradients. Bands were collected diluted in PBS supplemented with 1% FBS, and virus titers were determined by standard plaque assay on C7/10 cells [44].

### RNA labeling and electrophoresis

C7/10 cells were infected with EIL/GFP/VEEV at an MOI of 20 PFU/cell and incubated at 30°C for 16 h. Viral RNAs were then labeled with [^3^H]uridine (50 uCi/ml) between 16 and 24 h post infection in the presence of 1 μg/ml of Actinomycin D. RNAs were isolated from the cells and viral particles recovered from the supernatant by ultracentrifugation through 25% sucrose, and analyzed by agarose gel electrophoresis under denaturing conditions [69].

### RT-qPCR

For the analysis of intracellular concentration of virus-specific RNAs, relative concentration of IFIT1-specific mRNA, and analysis of RNA content in the released viral particles, RNA were isolated using the RNeasy minikit (Qiagen). cDNA were synthesized using the QuantiTect reverse transcription kit (Qiagen). Quantitative PCR was performed using the SsoFast EvaGreen Supermix (Bio-Rad) in a CFX96 real-time PCR detection system (Bio-Rad) for 40 cycles. The specificity of the quantitative PCR was confirmed by analyzing the melting temperatures of the amplified products. The efficiency of each pair of primers was determined using the standard curves obtained by performing real-time PCR on 10-fold dilutions of a control sample. For RNA samples isolated from the cells, the qPCR reactions were performed in parallel with primers specific to β-actin for normalization, and the fold difference in RNA concentration was calculated using the ΔΔ*CT* method. Each qPCR was performed in triplicate, and the means and standard deviations were calculated.

For RNAs isolated from viral particles, RT-qPCR was performed as described above using primers specific to the EILV nsP region, GFP, or VEEV E2 gene in parallel. The absolute copy number of each amplicon was determined based on a standard curve obtained using cDNA from *in vitro* synthesized EIL/GFP/VEEV genomic RNA, containing a known amount of RNA copies. The number of genomic RNA copies was obtained with the EILV-nsP primers. The number of SG RNA 1 copies represents the number of copies obtained determined with GFP primers after subtraction of the number of G RNA copies. Finally, the number of SG RNA 2 represents the number of copies determined using the primers specific to the structural E2 gene with subtraction of the number of copies determined by using GFP-specific primers.

### Analysis of virus replication

NIH 3T3, MEFs or C7/10 cells were seeded into 6-well Costar plates and infected at MOIs indicated in the figure legends. After 1 h incubation at 37°C (for NIH 3T3 and MEFs) or 30°C (for C7/10), cells were washed twice with PBS, overlaid with 1 ml of complete media and further incubated at the cell-specific temperatures. At the indicated times post infection, media were replaced. Virus titers in the harvested samples were determined by standard plaque assay on BHK-21 or C7/10 cells, as indicated in the figure legends.

### IFIT1-expressing stable cell lines

The IFIT1-encoding ORF was synthesized by RT-PCR from RNA isolated from NIH 3T3 cells, which were pre-treated with 500 IU/ml of IFN-β for 24 h. It was cloned into a modified PiggyBAC plasmid under control of the CMV promoter. MEF and IFIT locus^-/-^ MEF (3 x 10^5^ cells/well) were seeded in 6-well plates and transfected with the plasmid using the TransIT-3T3 kit according to the manufacturer’s instructions (Mirus). After blasticidin selection, cells were either used as a pool or further cloned to isolate clones with different levels of IFIT1 expression. Three clones, IFIT1 KI/1, IFIT1 KI/2 and IFIT1 KI/3, demonstrating different levels of IFIT1 by qPCR and Western blot, were used for further experiments.

### Luciferase assay

NIH-3T3 cells or MEFs in the 6-well Costar plates were either treated with IFN-β or remained mock-treated as described in the figure legends. Cells were infected with EIL/5’nLuc/VEEV variants at an MOI of 10 PFU/cell. For the viruses encoding nLuc with a modified 5’UTR, in some cases, MOIs were adjusted to obtain equivalent amounts of nLuc activity in the mock-treated, infected cells. Cells were infected for 1 h (see figure legends), washed with PBS and overlaid with 1 ml of complete media. At the specified time points, cells were lysed in reporter lysis buffer (Promega) and frozen at -80°C. After thawing, cell lysates were clarified by centrifugation for 1 min at 16,000 x g, and nLuc activity was determined using the Nano-Glo luciferase assay system (Promega) according to the manufacturer’s instructions. For all experiments, one of at least three independent and reproducible experiments is presented.

### Western blotting

The lysates of IFIT1 KI/1, IFIT1 KI/2, IFIT1 KI/3 and wt MEFs were treated overnight with 500 IU/ml of IFN-β, were analyzed by 10% SDS-PAGE. Proteins were transferred on nitrocellulose membrane and stained with mouse IFIT1-specific mAb (clone ISG56/13) followed by AlexaFluor800-labeled secondary Abs. Fluorescence intensities were analyzed and quantified on a LI-COR imager.

### Viral particles adsorption assay

NIH-3T3 cells were seeded in 8-well Ibidi chambers, and either treated or mock-treated with 100 IU/ml of IFN-β for 20 h at 37°C. To allow viral particles adsorption without internalization, cells were incubated with VEEV TC-83 for 1 h at 4°C, washed with cold PBS and immediately fixed with 4% paraformaldehyde (PFA). Adsorbed viral particles were stained using VEEV TC-83-specific mouse Abs (gift of Robert Tesh, UTMB) and AlexaFluor555-labeled secondary Abs. Cell nuclei were stained with Hoechst dye. Images were acquired on a Zeiss LSM700 confocal microscope with a 63X 1.4NA PlanApochromat oil objective. The 3D image stacks were further processed using Huygens Professional (Scientific Volume Imaging, Hilversum, Netherlands) for deconvolution, using experimental PSF, and Imaris for 3D rendering (Bitplane AG, St. Paul, MN). Spot function of Imaris was used for quantitative analysis of the numbers of attached virions.

### Virus entry and uncoating analysis

NIH-3T3 cells were seeded into 8-well Ibidi chambers and either treated or mock-treated with 100 IU/ml of IFN-β for 20 h. Cells were incubated with concentrated virus for 1 h at 4°C to allow virus adsorption to the cells, then washed and incubated for 1 h to 37°C in medium supplemented with 50 μg/ml of cycloheximide to allow entry and nucleocapsid disassembly in the absence of translation and replication of the incoming viral RNAs. Cells were then fixed with 4% PFA and stained using a rat mAb specific to the amino-terminal fragment of capsid protein, which is not exposed in assembled nucleocapsids, and AlexaFluor555-labeled secondary Abs. Nuclei were stained with Hoechst dye. Images were acquired on a Zeiss LSM700 confocal microscope with a 63X 1.4NA PlanApochromat oil objective. Images were assembled in Imaris (Bitplane AG, St. Paul, MN).

### IFN-β ELISA

Concentrations of IFN-β in the culture supernatants of infected cells were quantified using the VeriKine Mouse IFN Beta enzyme-linked immunosorbent assay (ELISA) kit (PBL Interferon Source) according to the manufacturer’s instructions.

### Microarray analysis

NIH 3T3 cells and IFN-α/βR^-/-^ MEFs were infected with VEEV/GFP/C1 at an MOI of 20 PFU/cell or treated with 1000 IU/ml of IFN-β. At the indicated time points, total RNA was isolated using TRIzol according to the manufacturer’s instructions (Invitrogen) and additionally purified with the RNeasy minikit (Qiagen). The cDNA synthesis, labeling, hybridization on Mouse Gene 1.0 ST Array GeneChips (Affymetrix) and image processing were performed at the Heflin Center Genomics Core facility (UAB). Two independent RNA samples were prepared for each indicated time point for VEEV/GFP/C1-infected cells and three RNA samples for IFN-β-treated cells. The robust multichip average (RMA) algorithm was used to normalize the raw intensity values using the GeneSpring software program, version GX 11.5 (Agilent).

### Animal studies

To assess the residual virulence of the designed variant of VEEV TC-83, 5’mutVEEV, 6-day-old NIH Swiss mice (Harlan) were inoculated subcutaneously (s.c.) with 2x10^5^ or 10^6^ PFU of the viruses diluted in PBS in a volume of 20 μl. Animals were checked twice daily for signs of the disease or death. Weight was evaluated on a daily basis.

### Ethics statement

The animal studies were carried out in accordance with the recommendations in the Guide for the Care and Use of Laboratory Animals of the National Institutes of Health. The protocol was approved by the Institutional Animal Care and Use Committee of the University of Alabama at Birmingham (Project Number: 09469).

## Supporting Information

S1 Fig Analysis of VEEV TC-83 and EIL/VEEV replication in NIH 3T3 cells. **(A)** Schematic representation of viral genomes. **(B)** NIH 3T3 and C7/10 cells were infected with VEEV TC-83 at an MOI of 10 PFU/cell. Media were harvested at the indicated times post infection, and virus titers were determined by plaque assay on BHK-21 cells. **(C)** NIH 3T3 cells were infected with VEEV TC-83 and EIL/VEEV at an MOI of 20 PFU/cell. Cells were harvested at the indicated times post infection, and RNAs were isolated as described in Materials and Methods. Numbers of RNAs copies in the samples were determined by RT-qPCR using VEEV E2-specific primers. They were normalized to concentration of β-acting mRNA and then to concentration of VEEV E2 gene-containing RNA in the samples harvested after virus adsorption to the cells.
(TIF)

S2 Fig IFN-β pretreatment inhibits GFP expression from subgenomic RNA, delivered by EIL/GFP/VEEV viral particles. NIH 3T3 cells were either treated with 500 IU/ml of IFN-β or mock-treated and then infected with EIL/GFP/VEEV at an MOI of 20 PFU/cell. GFP expression was evaluated at 4 h post infection.
(TIF)

S3 Fig Expression of nLuc increases for at least 24 h in C7/10, but not NIH 3T3, cells. NIH 3T3 and C7/10 cells were infected at the same MOI with EIL/nLuc/VEEV. Cells were harvested at the indicated time points, and nLuc activities were assessed. Data were normalized to nLuc activities measured after virus adsorption to the cells.
(TIF)

S4 Fig Binding of EIL/nLuc/VEEV to NIH 3T3 cells. NIH 3T3 cells were incubated with indicated doses of purified EIL/nLuc/VEEV for 1 h at 4^°^C. After washing, cells were lysed and nLuc activity was assessed.
(TIF)

S5 Fig Computer-predicted (m-Fold) secondary structures of the 5’UTRs used in translation experiments and the recombinant viruses, designed in this study. The introduced or identified mutations are indicated in red.
(TIF)

S6 Fig Analysis of IFIT1 expression in selected clones of wt IFIT1 KI MEFs and IFN-β-treated MEFs by Western blot. Data were normalized to β-tubulin levels and the expression level of IFIT1 detected in mock-treated wt MEFs.
(TIF)
