# Supplementary figures and images for: IFIT1 Differentially Interferes with Translation and Replication of Alphavirus Genomes and Promotes Induction of Type I Interferon
Source: PLoS Pathog. 2015 Apr 30;11(4):e1004863. doi: 10.1371/journal.ppat.1004863 (PMC4415776; doi:10.1371/journal.ppat.1004863)

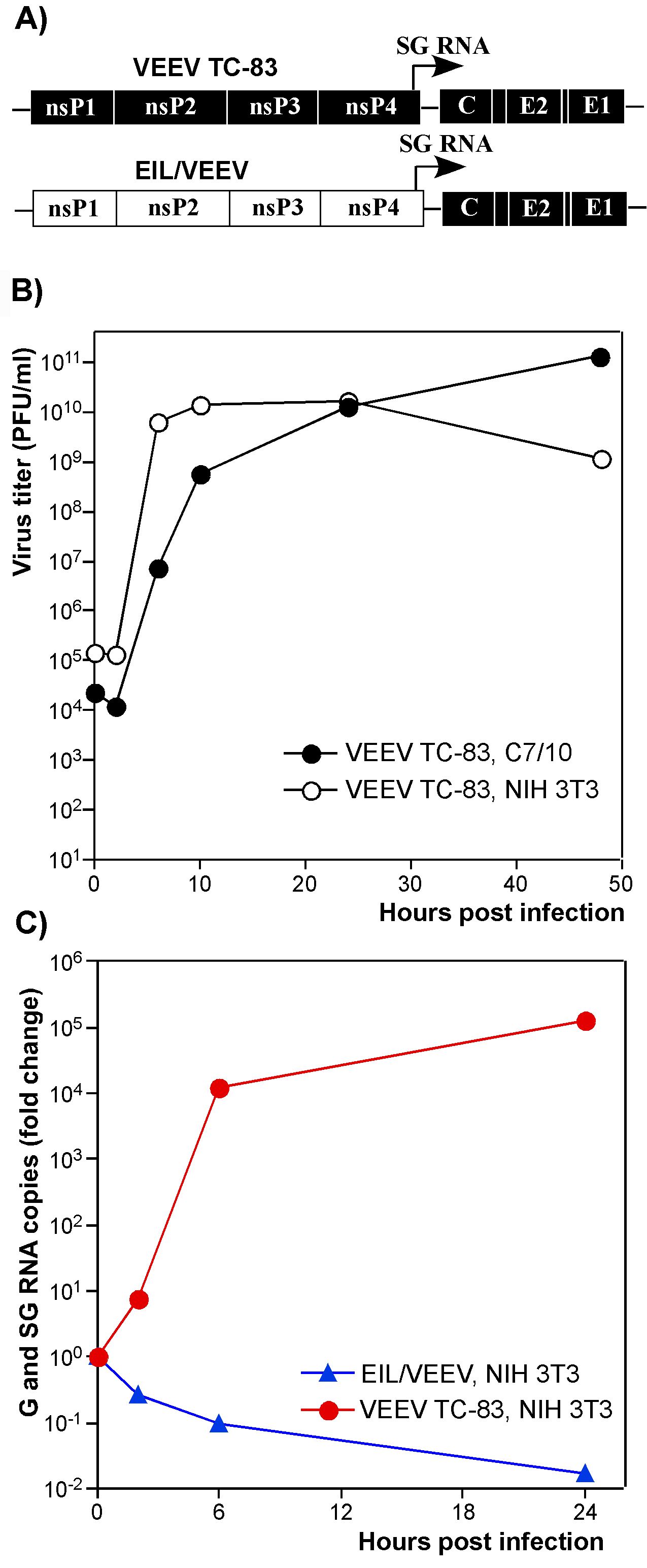

Supplement: S1 Fig — (A) Schematic representation of viral genomes. (B) NIH 3T3 and C7/10 cells were infected with VEEV TC-83 at an MOI of 10 PFU/cell. Media were harvested at the indicated times post infection, and virus titers were determined by plaque assay on BHK-21 cells. (C) NIH 3T3 cells were infected with VEEV TC-83 and EIL/VEEV at an MOI of 20 PFU/cell. Cells were harvested at the indicated times post infection, and RNAs were isolated as described in Materials and Methods. Numbers of RNAs copies in the samples were determined by RT-qPCR using VEEV E2-specific primers. They were normalized to concentration of β-acting mRNA and then to concentration of VEEV E2 gene-containing RNA in the samples harvested after virus adsorption to the cells. (TIF) [file ppat.1004863.s001.tif]

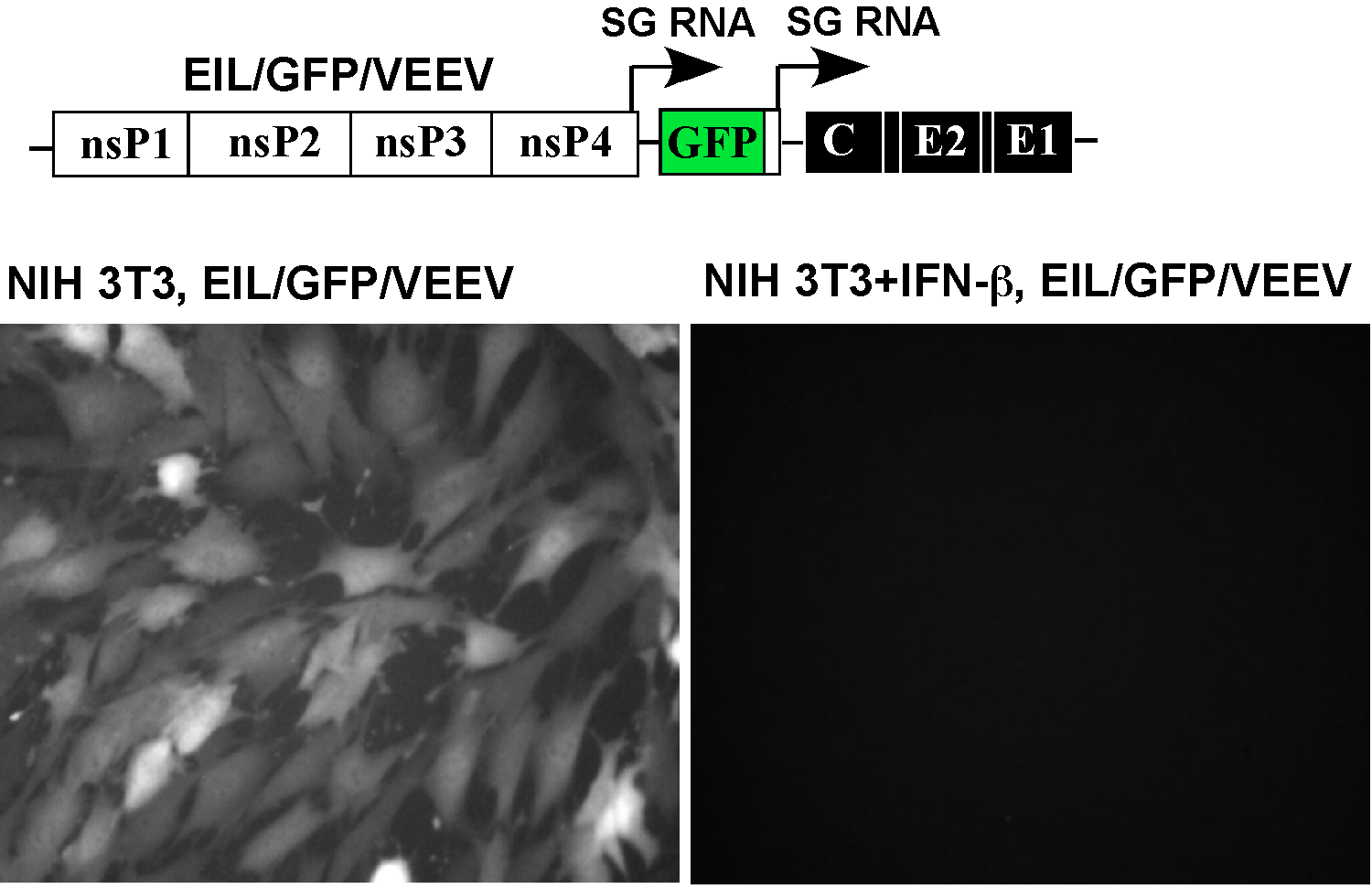

Supplement: S2 Fig — NIH 3T3 cells were either treated with 500 IU/ml of IFN-β or mock-treated and then infected with EIL/GFP/VEEV at an MOI of 20 PFU/cell. GFP expression was evaluated at 4 h post infection. (TIF) [file ppat.1004863.s002.tif]

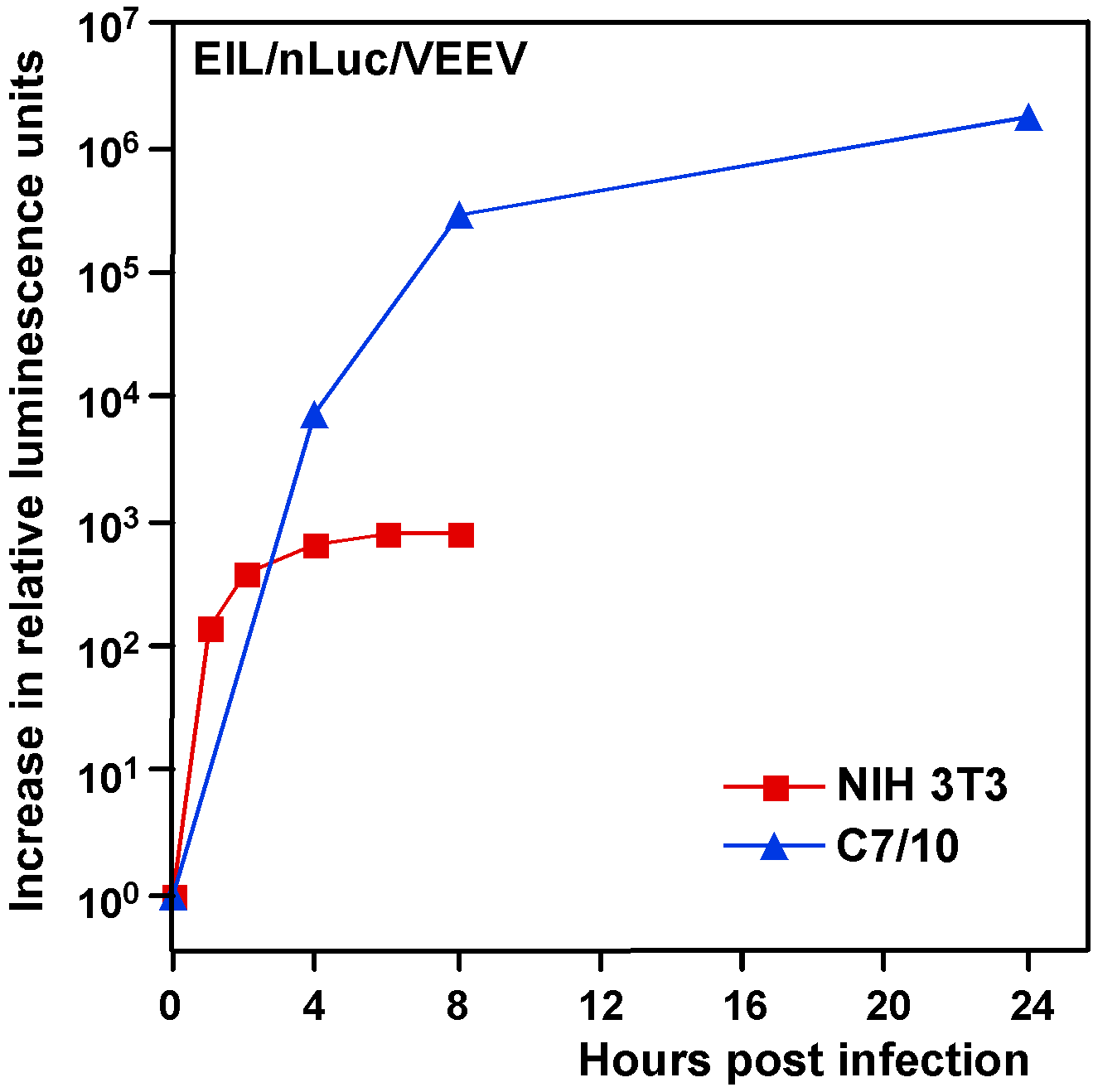

Supplement: S3 Fig — NIH 3T3 and C7/10 cells were infected at the same MOI with EIL/nLuc/VEEV. Cells were harvested at the indicated time points, and nLuc activities were assessed. Data were normalized to nLuc activities measured after virus adsorption to the cells. (TIF) [file ppat.1004863.s003.tif]

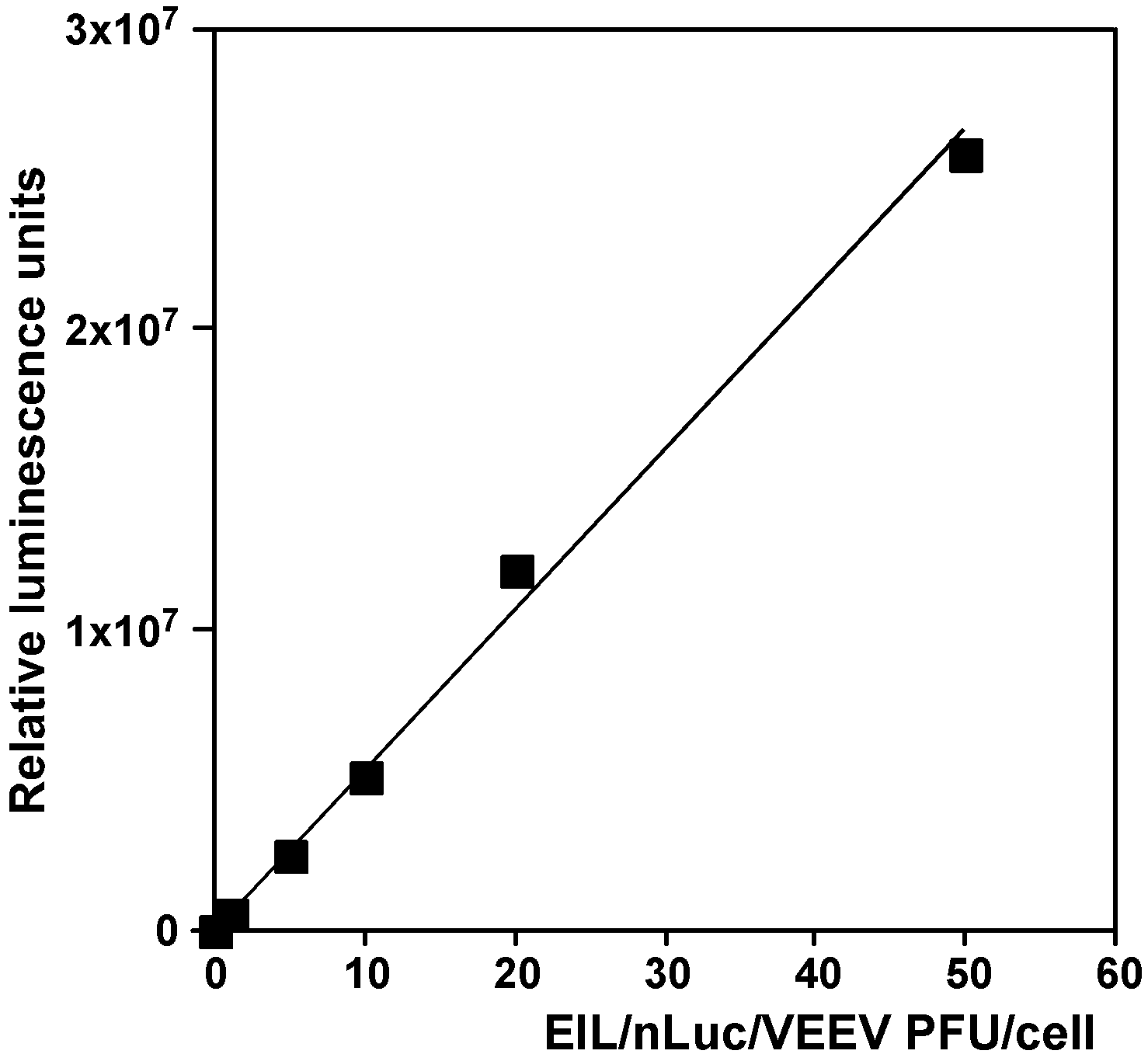

Supplement: S4 Fig — NIH 3T3 cells were incubated with indicated doses of purified EIL/nLuc/VEEV for 1 h at 4°C. After washing, cells were lysed and nLuc activity was assessed. (TIF) [file ppat.1004863.s004.tif]

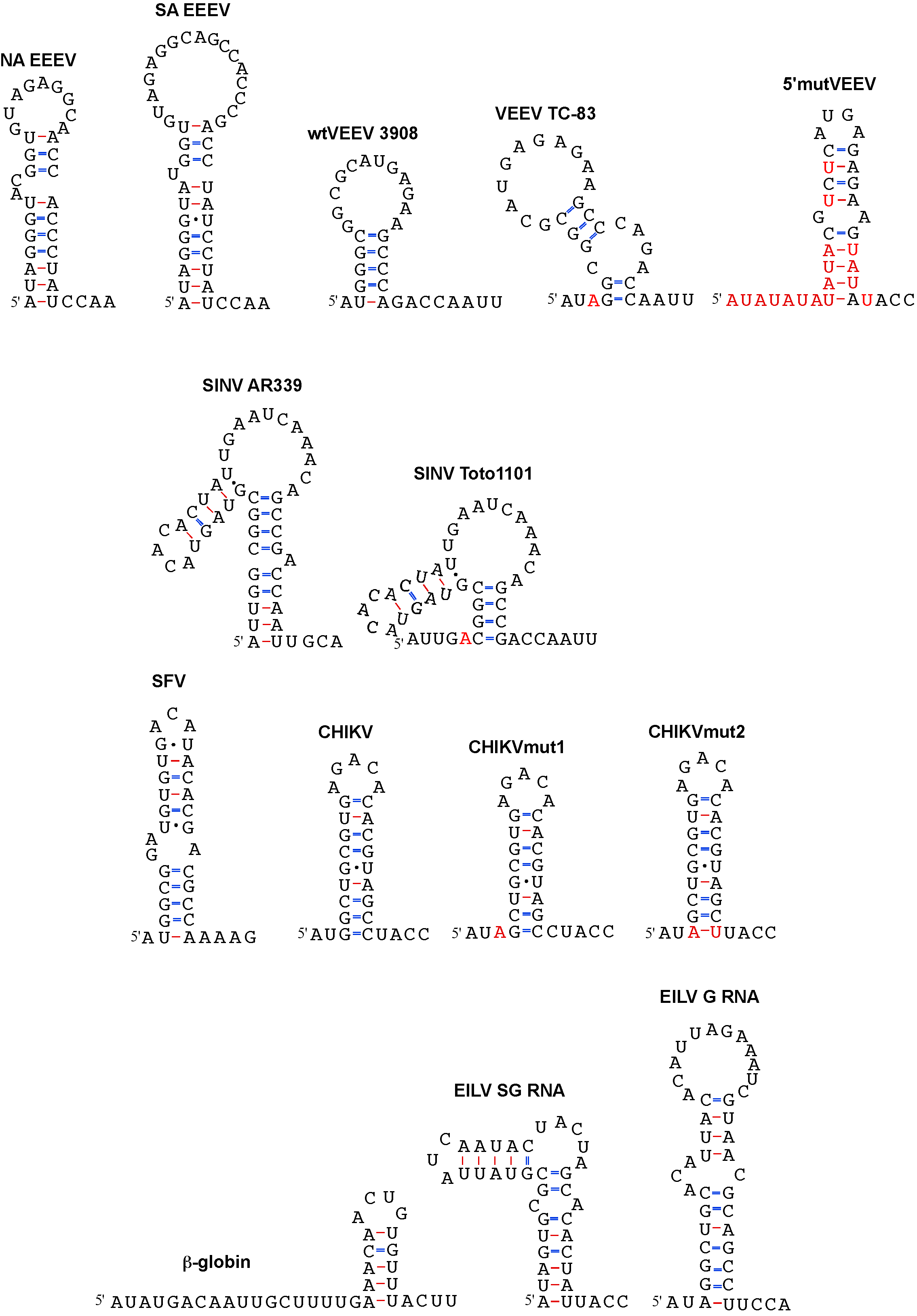

Supplement: S5 Fig — The introduced or identified mutations are indicated in red. (TIF) [file ppat.1004863.s005.tif]

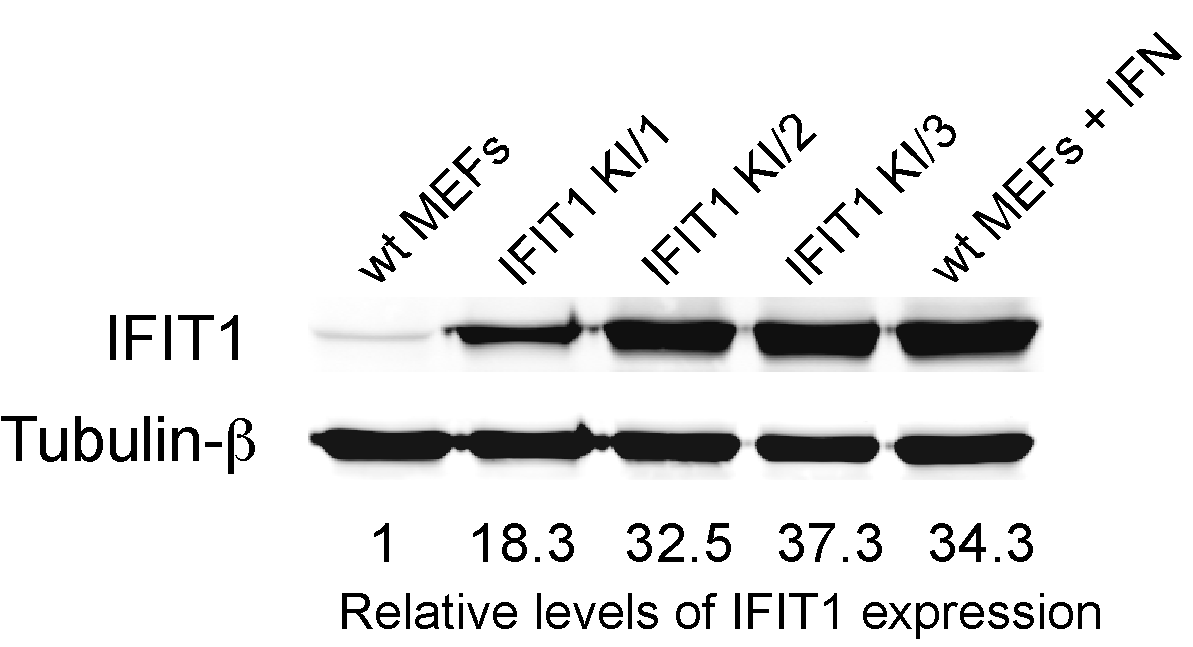

Supplement: S6 Fig — Data were normalized to β-tubulin levels and the expression level of IFIT1 detected in mock-treated wt MEFs. (TIF) [file ppat.1004863.s006.tif]
